# Early Release Science of the exoplanet WASP-39b with JWST NIRISS

**DOI:** 10.1038/s41586-022-05674-1

**Published:** 2023-01-09

**Authors:** Adina D. Feinstein, Michael Radica, Luis Welbanks, Catriona Anne Murray, Kazumasa Ohno, Louis-Philippe Coulombe, Néstor Espinoza, Jacob L. Bean, Johanna K. Teske, Björn Benneke, Michael R. Line, Zafar Rustamkulov, Arianna Saba, Angelos Tsiaras, Joanna K. Barstow, Jonathan J. Fortney, Peter Gao, Heather A. Knutson, Ryan J. MacDonald, Thomas Mikal-Evans, Benjamin V. Rackham, Jake Taylor, Vivien Parmentier, Natalie M. Batalha, Zachory K. Berta-Thompson, Aarynn L. Carter, Quentin Changeat, Leonardo A. dos Santos, Neale P. Gibson, Jayesh M. Goyal, Laura Kreidberg, Mercedes López-Morales, Joshua D. Lothringer, Yamila Miguel, Karan Molaverdikhani, Sarah E. Moran, Giuseppe Morello, Sagnick Mukherjee, David K. Sing, Kevin B. Stevenson, Hannah R. Wakeford, Eva-Maria Ahrer, Munazza K. Alam, Lili Alderson, Natalie H. Allen, Natasha E. Batalha, Taylor J. Bell, Jasmina Blecic, Jonathan Brande, Claudio Caceres, S. L. Casewell, Katy L. Chubb, Ian J. M. Crossfield, Nicolas Crouzet, Patricio E. Cubillos, Leen Decin, Jean-Michel Désert, Joseph Harrington, Kevin Heng, Thomas Henning, Nicolas Iro, Eliza M.-R. Kempton, Sarah Kendrew, James Kirk, Jessica Krick, Pierre-Olivier Lagage, Monika Lendl, Luigi Mancini, Megan Mansfield, E. M. May, N. J. Mayne, Nikolay K. Nikolov, Enric Palle, Dominique J. M. Petit dit de la Roche, Caroline Piaulet, Diana Powell, Seth Redfield, Laura K. Rogers, Michael T. Roman, Pierre-Alexis Roy, Matthew C. Nixon, Everett Schlawin, Xianyu Tan, P. Tremblin, Jake D. Turner, Olivia Venot, William C. Waalkes, Peter J. Wheatley, Xi Zhang

**Affiliations:** 1 Department of Astronomy and Astrophysics, University of Chicago, Chicago, IL USA; 2 Department of Physics, Université de Montréal, Montreal, Quebec Canada; 3 Institute for Research on Exoplanets, Université de Montréal, Montreal, Quebec Canada; 4 School of Earth and Space Exploration, Arizona State University, Tempe, AZ USA; 5 Department of Astrophysical and Planetary Sciences, University of Colorado Boulder, Boulder, CO USA; 6 Department of Astronomy and Astrophysics, University of California, Santa Cruz, Santa Cruz, CA USA; 7 Space Telescope Science Institute, Baltimore, MD USA; 8 Department of Physics & Astronomy, Johns Hopkins University, Baltimore, MD USA; 9 Earth and Planets Laboratory, Carnegie Institution for Science, Washington, DC USA; 10 Department of Earth and Planetary Sciences, Johns Hopkins University, Baltimore, MD USA; 11 Department of Physics and Astronomy, University College London, London, UK; 12 INAF - Osservatorio Astrofisico di Arcetri, Florence, Italy; 13 School of Physical Sciences, The Open University, Milton Keynes, UK; 14 Division of Geological and Planetary Sciences, California Institute of Technology, Pasadena, CA USA; 15 Department of Astronomy, University of Michigan, Ann Arbor, MI USA; 16 Department of Astronomy, Cornell University, Ithaca, NY USA; 17 Carl Sagan Institute, Cornell University, Ithaca, NY USA; 18 Max Planck Institute for Astronomy, Heidelberg, Germany; 19 Department of Earth, Atmospheric and Planetary Sciences, Massachusetts Institute of Technology, Cambridge, MA USA; 20 Kavli Institute for Astrophysics and Space Research, Massachusetts Institute of Technology, Cambridge, MA USA; 21 Atmospheric, Oceanic and Planetary Physics, Department of Physics, University of Oxford, Oxford, UK; 22 Observatoire de la Côte d’Azur, CNRS, Laboratoire Lagrange, Université Côte d’Azur, Nice, France; 23 Astrobiology Program, UC Santa Cruz, Santa Cruz, CA USA; 24 European Space Agency, Space Telescope Science Institute, Baltimore, MD USA; 25 School of Physics, Trinity College Dublin, Dublin, Ireland; 26 School of Earth and Planetary Sciences (SEPS), National Institute of Science Education and Research (NISER), Homi Bhabha National Institute (HBNI), Jatani, India; 27 Center for Astrophysics | Harvard & Smithsonian, Cambridge, MA USA; 28 Department of Physics, Utah Valley University, Orem, UT USA; 29 Leiden Observatory, University of Leiden, Leiden, The Netherlands; 30 SRON Netherlands Institute for Space Research, Leiden, The Netherlands; 31 Universitäts-Sternwarte, Ludwig-Maximilians-Universität München, Munich, Germany; 32 Exzellenzcluster Origins, Garching, Germany; 33 Lunar and Planetary Laboratory, University of Arizona, Tucson, AZ USA; 34 Instituto de Astrofísica de Canarias (IAC), Tenerife, Spain; 35 Departamento de Astrofísica, Universidad de La Laguna (ULL), Tenerife, Spain; 36 INAF - Palermo Astronomical Observatory, Palermo, Italy; 37 Johns Hopkins University Applied Physics Laboratory, Laurel, MD USA; 38 School of Physics, University of Bristol, Bristol, UK; 39 Centre for Exoplanets and Habitability, University of Warwick, Coventry, UK; 40 Department of Physics, University of Warwick, Coventry, UK; 41 NASA Ames Research Center, Moffett Field, CA USA; 42 Bay Area Environmental Research Institute, NASA Ames Research Center, Moffett Field, CA USA; 43 Department of Physics, New York University Abu Dhabi, Abu Dhabi, United Arab Emirates; 44 Center for Astro, Particle, and Planetary Physics (CAP3), New York University Abu Dhabi, Abu Dhabi, United Arab Emirates; 45 Department of Physics & Astronomy, University of Kansas, Lawrence, KS USA; 46 Instituto de Astrofísica, Universidad Andrés Bello, Santiago, Chile; 47 Núcleo Milenio de Formación Planetaria (NPF), Valparaíso, Chile; 48 Centro de Astrofísica y Tecnologías Afines (CATA), Santiago, Chile; 49 School of Physics and Astronomy, University of Leicester, Leicester, UK; 50 Centre for Exoplanet Science, University of St Andrews, St Andrews, UK; 51 INAF - Osservatorio Astrofisico di Torino, Pino Torinese, Italy; 52 Space Research Institute, Austrian Academy of Sciences, Graz, Austria; 53 Institute of Astronomy, Department of Physics and Astronomy, KU Leuven, Leuven, Belgium; 54 Anton Pannekoek Institute for Astronomy, University of Amsterdam, Amsterdam, The Netherlands; 55 Planetary Sciences Group, Department of Physics, University of Central Florida, Orlando, FL USA; 56 Florida Space Institute, University of Central Florida, Orlando, FL USA; 57 ARTORG Center for Biomedical Engineering Research, University of Bern, Bern, Switzerland; 58 Institute for Astrophysics, University of Vienna, Vienna, Austria; 59 Department of Astronomy, University of Maryland, College Park, MD USA; 60 Department of Physics, Imperial College London, London, UK; 61 Infrared Processing and Analysis Center (IPAC), California Institute of Technology, Pasadena, CA USA; 62 Université Paris-Saclay, Université Paris Cité, CEA, CNRS, AIM, Gif-sur-Yvette, France; 63 Département d’Astronomie, Université de Genève Sauverny, Versoix, Switzerland; 64 Department of Physics, University of Rome “Tor Vergata”, Rome, Italy; 65 INAF - Turin Astrophysical Observatory, Pino Torinese, Italy; 66 Steward Observatory, University of Arizona, Tucson, AZ USA; 67 Department of Physics and Astronomy, Faculty of Environment, Science and Economy, University of Exeter, Exeter, UK; 68 Astronomy Department, Wesleyan University, Middletown, CT USA; 69 Van Vleck Observatory, Wesleyan University, Middletown, CT USA; 70 Institute of Astronomy, University of Cambridge, Cambridge, UK; 71 Universidad Adolfo Ibáñez, Campus Peñalolén, Santiago, Chile; 72 Maison de la Simulation, CEA, CNRS, Université Paris-Sud, Université Versailles St Quentin, Université Paris-Saclay, Gif-sur-Yvette, France; 73 Université de Paris Cité and Université Paris-Est Creteil, CNRS, LISA, Paris, France; 74 Astrophysics & Planetary Sciences, University of Colorado Boulder, Boulder, CO USA; 75 Department of Earth and Planetary Sciences, University of California, Santa Cruz, Santa Cruz, CA USA

**Keywords:** Exoplanets, Exoplanets

## Abstract

The Saturn-mass exoplanet WASP-39b has been the subject of extensive efforts to determine its atmospheric properties using transmission spectroscopy^1–4^. However, these efforts have been hampered by modelling degeneracies between composition and cloud properties that are caused by limited data quality^5–9^. Here we present the transmission spectrum of WASP-39b obtained using the Single-Object Slitless Spectroscopy (SOSS) mode of the Near Infrared Imager and Slitless Spectrograph (NIRISS) instrument on the JWST. This spectrum spans 0.6–2.8 μm in wavelength and shows several water-absorption bands, the potassium resonance doublet and signatures of clouds. The precision and broad wavelength coverage of NIRISS/SOSS allows us to break model degeneracies between cloud properties and the atmospheric composition of WASP-39b, favouring a heavy-element enhancement (‘metallicity’) of about 10–30 times the solar value, a sub-solar carbon-to-oxygen (C/O) ratio and a solar-to-super-solar potassium-to-oxygen (K/O) ratio. The observations are also best explained by wavelength-dependent, non-grey clouds with inhomogeneous coverageof the planet’s terminator.

## Abstract (web summary)

The transmission spectrum of the exoplanet WASP-39b is obtained using observations from the Single-Object Slitless Spectroscopy mode of the Near Infrared Imager and Slitless Spectrograph instrument aboard the JWST.

## Main

**Extended Data Fig. 1:**
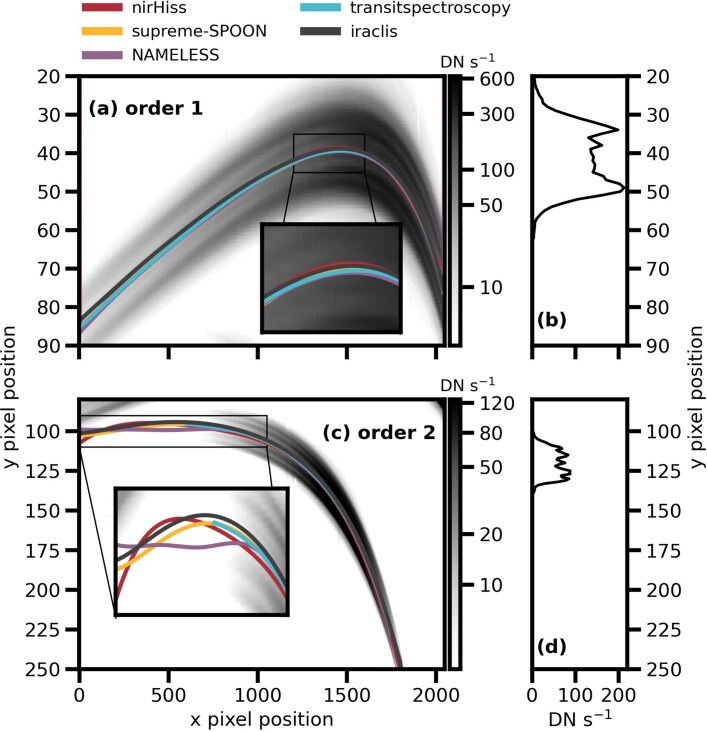
Comparison of the (*x*, *y*) positions of NIRISS orders 1 and 2 across the detector as modelled from different reduction pipelines. **a**,**c**, Each pipeline traces the curvature of orders 1 and 2 using different methods. We show the best-fit trace for order 1 in panel **a** and order 2 in panel **b**. We highlight zoomed-in regions to further examine differences. We note that iraclis uses the JWST-provided spectral trace. There is generally good agreement between the traces across the entire detector (<1 pixel deviations), with the strongest deviations towards the ends of each trace (for example, *x* pixel position <500 for orders 1 and 2). This demonstrates the reliability of order spectral traces across all pipelines. **b**,**d**, We provide an example spatial profile along the column for order 1 (**b**) and order 2 (**d**) at *x* = 1,250. (https://github.com/afeinstein20/wasp39b_niriss_paper/blob/main/scripts/edfigure1.py).

We observed a transit of WASP-39 b using the NIRISS^10^ on the JWST as part of the Transiting Exoplanet Community Early Release Science Program^11,12^. Our observations spanned 8.2 h starting on 26 July 2022 20:45 UTC, covering the 2.8-h transit as well as 3.0 h before and 2.4 h after the transit to establish a flux baseline. The data were taken in the SOSS mode, which simultaneously covers the wavelength range from 0.6 to 2.8 μm across two spectral orders on the same detector. Order 1 contains the spectral range between 0.6 and 2.8 μm at an average resolving power of *R* ≣ *λ*/Δ*λ* *=* 700, whereas order 2 delivers the spectral range of 0.6–1.4 μm at an average resolving power of *R* = 1,400. In the SOSS mode, the spectra are spread across more than 20 pixels in the cross-dispersion direction by means of a cylindrical defocusing lens (see Extended Data Fig. 1), thus allowing longer integration times and reducing the impact of pixel-level differences in the detector response. However, this defocus results in the physical overlap of both orders on the detector. The time-series observation was composed of 537 integrations of 49.4 s (nine groups per integration), corresponding to a duty cycle of 89%.

**Fig. 1:**
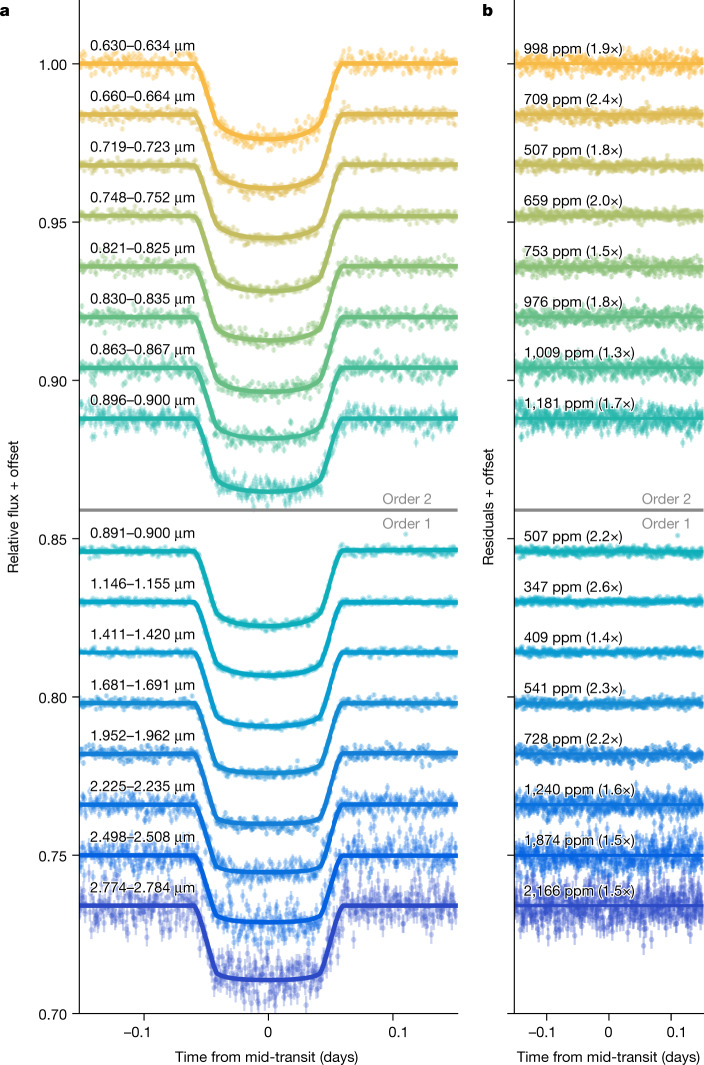
Selection of systematics-corrected spectrophotometric light curves and residuals for the transit of WASP-39b observed with NIRISS/SOSS for orders 1 and 2. An exoplanet transit model (solid line) was fitted to each light curve with chromatic_fitting using a quadratic limb-darkening law. The limb-darkening coefficients, planet-to-star radius ratio (*R*_p_/R_*_) and out-of-transit flux were varied in each wavelength channel, whereas all other parameters were fixed. The residuals to the best-fit models are shown for each light curve. The wavelength range for each channel is denoted in panel **a**, whereas parts-per-million (ppm) scatter in the residuals is denoted in panel **b**. We calculate the ppm as the standard deviation of the out-of-transit residuals. We quote the ratio of the predicted photon noise for each bin in brackets. The reductions are from the nirHiss and chromatic_fitting routines described in Methods. We define our errors as the 1*σ* uncertainties extracted from the stellar spectra. (https://github.com/afeinstein20/wasp39b_niriss_paper/blob/main/scripts/figure1.py).

**Fig. 2:**
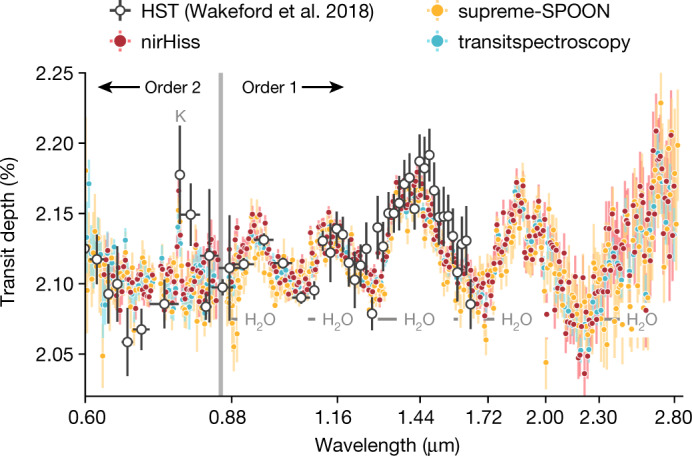
NIRISS transmission spectra for WASP-39b obtained by three data-reduction pipelines. We find broad agreement in the overall structure of the transmission spectra between several reduction pipelines, a sample of which are presented here (see Extended Data Fig. 5 for all reductions). The JWST data are shown in the coloured points, whereas previous HST observations of WASP-39b (ref. ^18^) are shown in white. We note that we only consider wavelengths <0.85 μm for order 2, as order 1 has much higher fidelity in the overlapping 0.85–1.0-μm range. We define our errors as the 1*σ* uncertainties extracted from the 16th and 84th percentiles of the transit depths fit from each pipeline. The JWST and HST data agree across the three broad H_2_O features that they have in common. We find evidence of a K absorption feature at 0.76 μm in the new JWST data, which was proposed in the previous HST data^18^. (https://github.com/afeinstein20/wasp39b_niriss_paper/blob/main/scripts/figure2.py).

**Extended Data Fig. 2:**
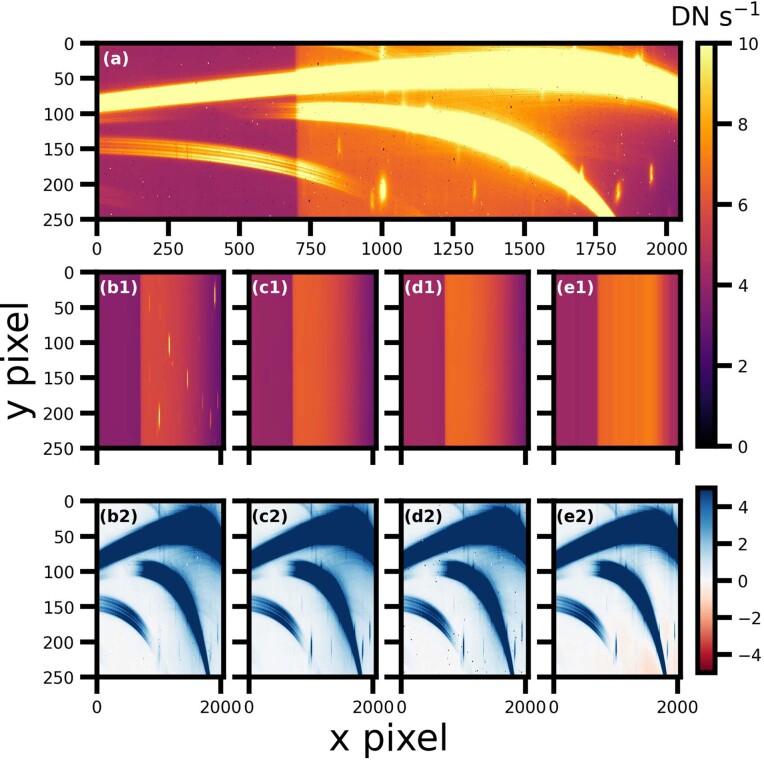
Comparison of averaged background frames computed for each reduction pipeline. **a**, A median out-of-transit integration from the stage 2 output files of the jwst pipeline in data numbers per second (DN s^−1^). **b1**,**c1**,**d1**,**e1**, Estimated median background frames for four example pipelines: nirHiss (**b**), supreme-SPOON (**c**), transitspectroscopy (**d**) and iraclis (**e**). All reduction pipelines use the predefined zodiacal background model provided on the STScI JDox User Documentation website. nirHiss estimates the zeroth-order contaminants by taking a smoothed median from the F277W filter integrations. We note that the background frame from supreme-SPOON (**c1**) for group eight is shown here and was scaled by a factor of about 0.02 to lie on the same scale as the background from the other three pipelines. iraclis subtracts a median per column to remove further 1/*f* noise. **b2**,**c2**,**d2**,**e2**, Background-subtracted median integrations for each pipeline, plotted in DN s^−1^ but scaled from −5 to 5 to highlight subtle changes in the background. For these integrations, we define out of transit as integrations 1–200 and 400–518. (https://github.com/afeinstein20/wasp39b_niriss_paper/blob/main/scripts/edfigure2.py).

**Extended Data Fig. 3:**
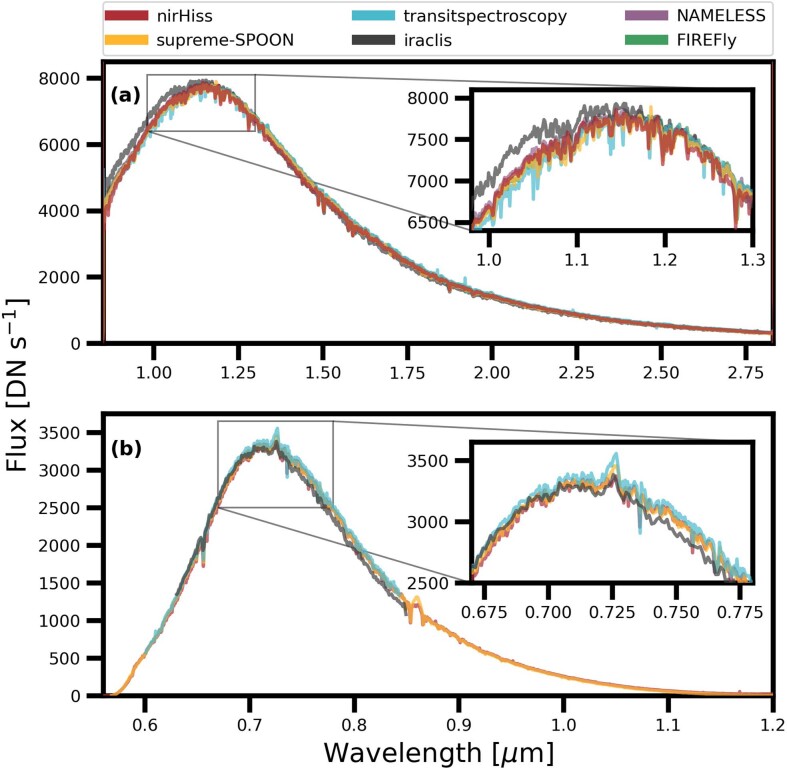
Example extracted stellar spectra from different reduction pipelines. The insets highlight the peak of the spectra. The supreme-SPOON spectra are scaled by a factor of 72 to compare the overall shape of the spectra, rather than the extracted flux counts. **a**, The extracted stellar spectra from order 1. All pipelines are in relatively good agreement, whereas the shape of the iraclis data changes slightly at *λ* < 1.1 μm. This is probably owing to different traces that were used in the spectral extraction routine. **b**, The extracted stellar spectra from order 2. Differences at *λ* = 0.725 and 0.860 μm are because of differences in removing zeroth-order contaminants in the background. The iraclis pipeline does not extract data past *λ* > 0.85 μm, which is where the order overlap region begins. Across all pipelines, the shape of the spectra, as well as overall absorption features, cosmic-ray-removal techniques and noise levels are consistent. (https://github.com/afeinstein20/wasp39b_niriss_paper/blob/main/scripts/edfigure3.py).

**Extended Data Fig. 4:**
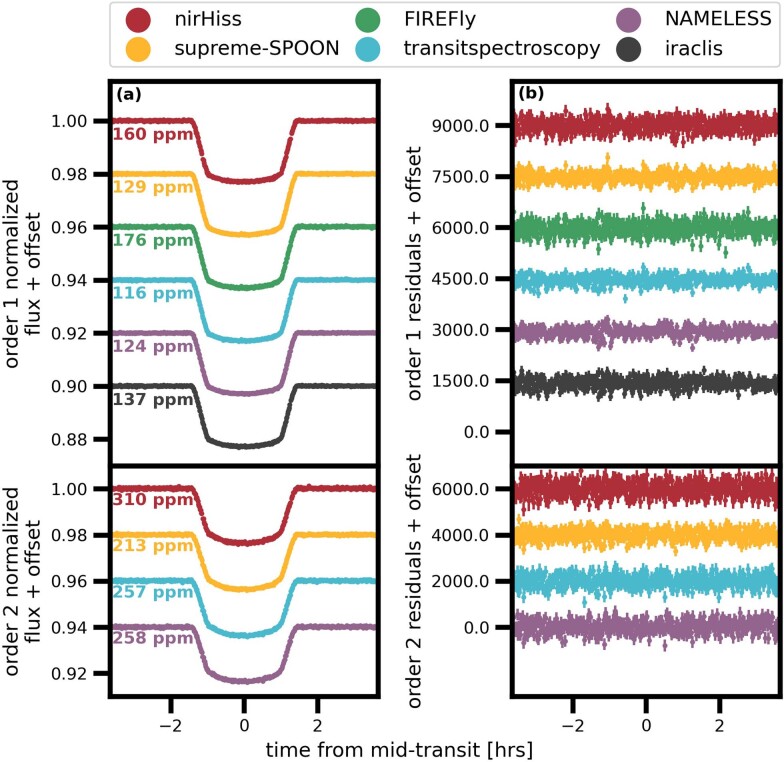
White-light curves and residuals between the light curve and best-fit exoplanet transit model for each reduction pipeline. **a**, White-light curves for order 1 (top) and order 2 (bottom) with the out-of-transit scatter noted in the figure text. **b**, The residuals, in ppm, between the plotted light curves and best-fit exoplanet transit model. The start of transit ingress and end of transit egress are marked with dashed vertical lines; the transit midpoint is marked with a solid vertical line. (https://github.com/afeinstein20/wasp39b_niriss_paper/blob/main/scripts/edfigure4.py).

**Extended Data Fig. 5:**
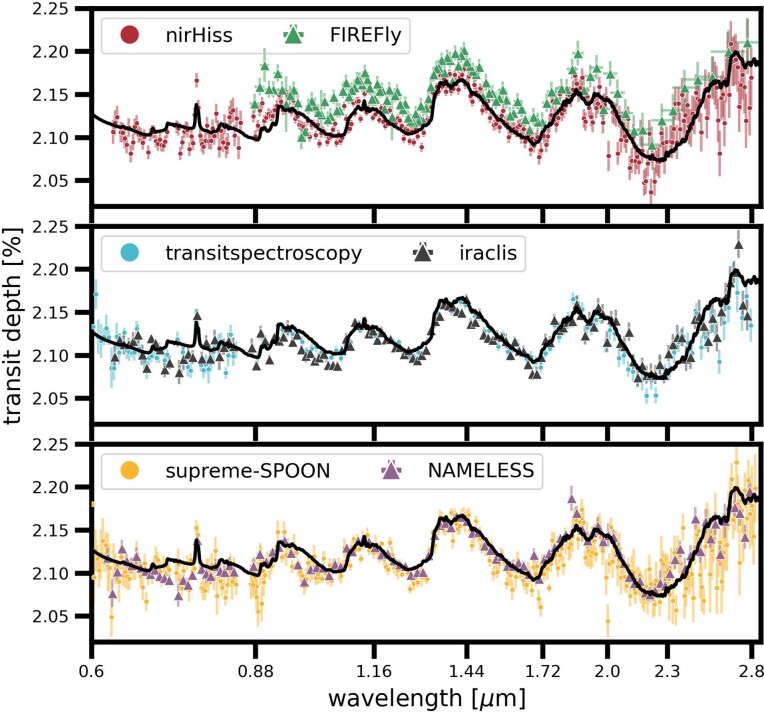
Transmission spectra for WASP-39b for all reduction techniques. Our best-fit reference model to the nirHiss spectrum (red) is plotted as a solid black line in all panels and the spectra are separated into three panels for ease of reading. Wavelengths that overlap with zeroth-order contaminants are masked. (https://github.com/afeinstein20/wasp39b_niriss_paper/blob/main/scripts/edfigure5.py).

We extracted the stellar spectra from the time-series observations using six different pipelines to test the impact of differences in spectral order tracing, 1/*f* noise correction, background removal and spectrum extraction methodology (see Methods and Extended Data Figs. 2 and 3). We created spectrophotometric light curves for each pipeline (Fig. 1) and summed the data to create white-light curves per spectral order (Extended Data Fig. 4). The spectrophotometric and white-light curves are largely free of instrumental systematics except for a constant-rate linear trend in time and an exponential ramp effect within the first 15 min of the time series. The fitted transit depths were binned into 80 spectral wavelength changes in order 1 and 20 in order 2 to create transmission spectra at *R* ≈ 300. We present the spectra from the nirHiss, supreme-SPOON and transitspectroscopy reduction pipelines in Fig. 2. We find consistent results between the pipelines, with the derived spectra also being in agreement with previous Hubble Space Telescope (HST) observations (see also Extended Data Fig. 5).

We investigated the atmospheric properties of WASP-39b by comparing our measured transmission spectrum from the nirHiss pipeline to grids of one-dimensional, radiative–convective–thermochemical equilibrium models. These models explore the impact of atmospheric metallicity (M/H), carbon-to-oxygen ratio (C/O), potassium-to-oxygen ratio (K/O), heat redistribution (*f*) and cloud coverage on the transmission spectrum of the planet. We explored several cloud models ranging from parametric treatments^13,14^ to a droplet sedimentation model^15^ that calculates the vertical distributions of cloud mass mixing ratio and mean particle size from the balance between gravitational sedimentation and eddy diffusion of cloud particles. Using a Bayesian inference framework (see Methods), we compared these grids of models to the observations and inferred the ranges of M/H, C/O ratio, K/O ratio and *f* that best explain the data while marginalizing over different cloud treatments. WASP-39, the host star, has a metallicity equal to that of the Sun within measurement precision^16–19^, so we reference the atmospheric abundances of the planet to the solar pattern of elemental abundances^20^. We compared the grid spectra computed by various models (PICASO, ATMO, PHOENIX and ScCHIMERA) with an observational spectrum obtained from each data-reduction pipeline and obtained broadly consistent results on the inferred atmospheric properties. We report the results from the comparison between the nirHiss spectrum and ScCHIMERA grid that allows the most comprehensive treatments of cloud properties.

**Fig. 3:**
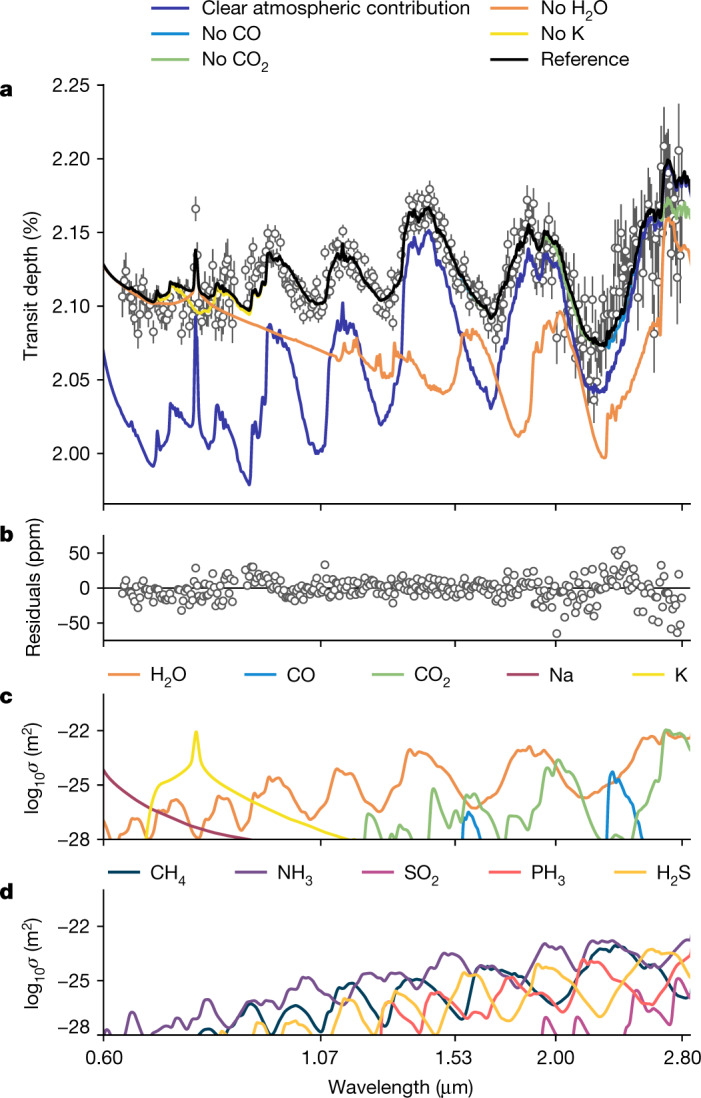
Interpretation of the constituents of the NIRISS WASP-39b transmission spectrum. **a**,**b**, Panel **a** shows the comparison of the transmission spectrum of WASP-39b from the nirHiss reduction (grey points) with respect to the best-fit reference model (black line). This model assumes an atmospheric metallicity of M/H = 1.38 (23 times the solar value), C/O ratio of 0.2 (0.55 times the solar value^20^), K/O ratio of 0.1 (1.26 times the solar value), full day–night heat redistribution (*f* = 1) and flux-balanced clouds with inhomogeneous terminator coverage. Each coloured line removes a key constituent found in our best-fit reference model to demonstrate how the spectrum would change were these features not included. The removal of clouds and H_2_O absorption from the reference model result in large-scale changes to the shape and depth of the transmission spectrum. Other sources of opacity with an impact on the spectrum are K, CO and CO_2_. Residuals between the data and the reference model are plotted in panel **b**. **c**,**d**, These two panels show the molecular absorption cross-sections for a selection of gases observable within the NIRISS bandpass. Panel **c** highlights gases inferred by our analysis of the spectrum of WASP-39b. Panel **d** highlights some gases that were not identified in these data but may be present in future observations of other exoplanets. We define our errors as the 1*σ* uncertainties extracted from the 16th and 84th percentiles of the transit depths fit from each pipeline. (https://github.com/afeinstein20/wasp39b_niriss_paper/blob/main/scripts/figure3.py).

Our best-fitting model to the NIRISS/SOSS transmission spectrum of WASP-39 b is presented in Fig. 3. The spectral maxima at 0.9, 1.15, 1.4 and 1.8 μm owing to water absorption result in a >30*σ* detection of the molecule (see Methods). Similarly, the potassium doublet at 0.768 μm is detected in the data at 6.8*σ*. Signatures of CO and/or CO_2_ are identified because of their contribution to the spectrum past 2.3 μm. We find a 3.6*σ* significance model preference for CO and no significant preference for CO_2_ (see Methods).

**Fig. 4:**
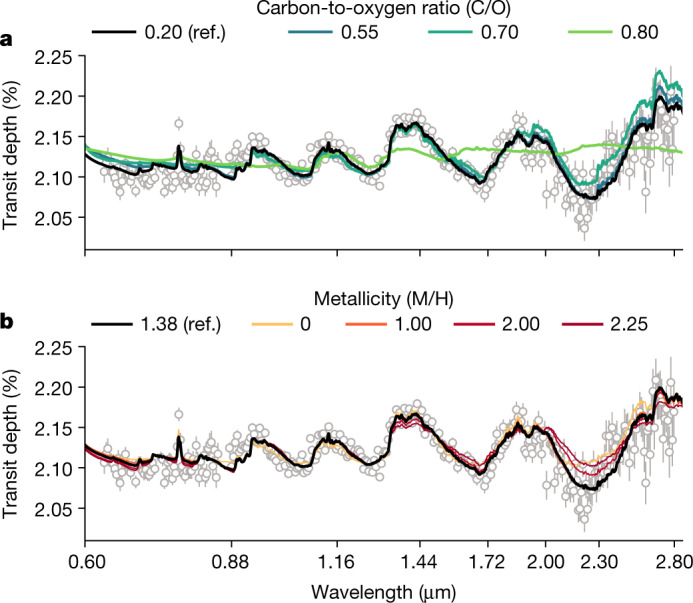
Impact of the C/O ratio and metallicity on the JWST-NIRISS spectrum of WASP-39b. **a**, Variation of the C/O ratio in the best-fit reference model while keeping the metallicity, redistribution and K/O ratio parameters from the reference model the same and fitting for the cloud parameters and scaled planetary radius to best explain the observations. Under these equilibrium conditions, increasing the C/O ratio results in less H_2_O and more CH_4_, the latter having spectroscopic signatures incompatible with the observations. To mute these incompatible CH_4_ features at high C/O ratios, the model requires a higher degree of cloudiness that also mutes any remaining H_2_O features in the spectrum. **b**, The same as for panel **a** but instead we vary the metallicity parameter. The metallicity constraint is driven by the *λ* > 2 μm data; the high-metallicity models (M/H > 2) expect larger transit depths than that seen in the data. The same reference model is plotted as a thick black line in both panels. We define our errors as the 1*σ* uncertainties extracted from the 16th and 84th percentiles of the transit depths fit from each pipeline. (https://github.com/afeinstein20/wasp39b_niriss_paper/blob/main/scripts/figure4.py).

From the chemical equilibrium models considered, we find that the observations are best explained by a sub-solar C/O ratio (see Fig. 4a). Across the different spectroscopic resolutions and atmospheric models, the best-fit C/O ratio is 0.2, which is the lowest ratio explored in the grid of models. We rule out super-solar C/O ratio because of the lack of CH_4_ features at about 1.7 μm and about 2.3 μm, at which they would be expected for C/O ratio ≳ 0.7. Overall, solar-to-super-solar C/O ratios fail to explain the transmission spectrum at the shortest (≲1 μm) and longest (≳2 μm) wavelengths. Our best-fit C/O ratio is broadly consistent with the observations of WASP-39b with NIRCam (2.4–4.0 μm; ref. ^21^), NIRSpec G395H (3–5 μm; ref. ^22^) and NIRSpec PRISM (0.5–5.0 μm; ref. ^23^).

We find that the observations are best explained by an atmospheric metallicity of 10–30 times solar. Metallicity inferences over the wavelength range of these observations are largely driven by the size and shape of the water-vapour features, with some minor contributions because of CO and/or CO_2_ at longer wavelengths (>2 μm; see Figs. 3 and 4b). The preferred range of metallicities provides the best fit to the shape and size of the muted water-vapour features shortward of 2 μm in combination with the larger water and CO/CO_2_ feature longward of 2 μm, regardless of the assumed cloud treatment in our models.

Owing to the simultaneous detection of potassium and water vapour, we are able to place constraints on the K/O ratio, which is a refractory-to-volatile elemental ratio, being a solar-to-super-solar value. Because the refractory elements are condensed into solids in most parts of protoplanetary disks, the disk gas accretion tends to cause a sub-stellar refractory elemental abundance^24^. By contrast, solid accretion, such as planetesimal accretion, acts to increase the refractory elemental abundance and refractory-to-volatile elemental ratio^25^, although the latter depends on the composition of the accreted solids^26^. We anticipate that the K/O ratio diagnoses to what degree the solid accretion enriched the atmosphere during the formation stage. All of our fitted models find that the WASP-39b observations are well described by solar-to-super-solar K/O ratios, which is in agreement with previous inferences for this planet obtained through observations with limited spectral coverage^27^. We do not expect the K feature to be affected by stellar chromospheric magnetic activity given the effective temperature of the star of approximately 5,300 K (ref. ^28^) and the general quietness of WASP-39 (see ref. ^21^). It is also in line with larger population studies of hot giant planets that broadly found solar-to-super-solar refractory abundances and solar-to-sub-solar H_2_O abundances^27,29^. The shape and strength of the potassium doublet are best explained by K/O ratios of 0.1–0.5, equivalent to 1–3 times solar (see Extended Data Fig. 8), whereas the suggested K/O ratio might be a lower limit owing to the photoionization of K at upper atmospheres^30^.

**Extended Data Fig. 6:**
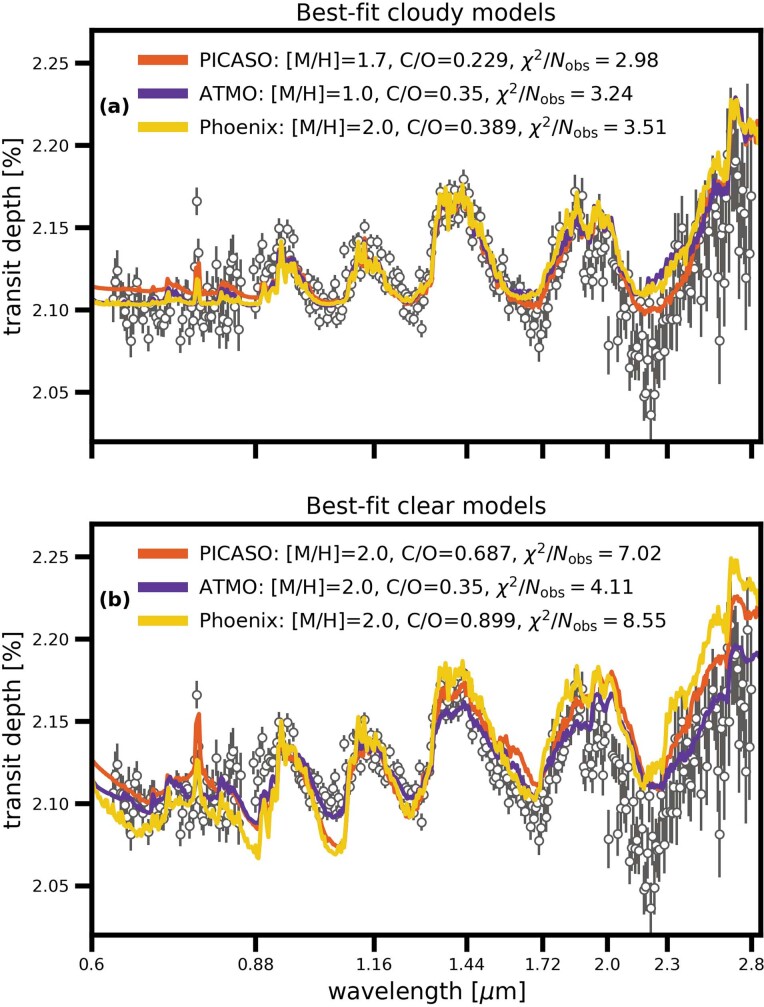
A summary of precomputed forward model fits to the NIRISS/SOSS spectrum. **a**, Each coloured line shows the best-fit spectrum from the PICASO, ATMO and PHOENIX cloudy grid. The *χ*^2^ values per number of data points (*N*_obs_ = 327) are *χ*^2^/*N*_obs_ = 2.98, 3.24 and 3.51 for the PICASO, ATMO and PHOENIX grids, respectively. All grid models consistently indicate a super-solar metallicity of M/H = 1–2 and a sub-solar C/O ratio. **b**, The same as the top panel but for the best-fit clear atmosphere models. The clear models yield noticeably worse fits to the data, *χ*^2^/*N*_obs_ = 7.02, 4.11 and 8.55 for the PICASO, ATMO and PHOENIX grids, respectively, which strongly indicates the presence of clouds in the atmosphere. (https://github.com/afeinstein20/wasp39b_niriss_paper/blob/main/scripts/edfigure6.py).

**Extended Data Fig. 7:**
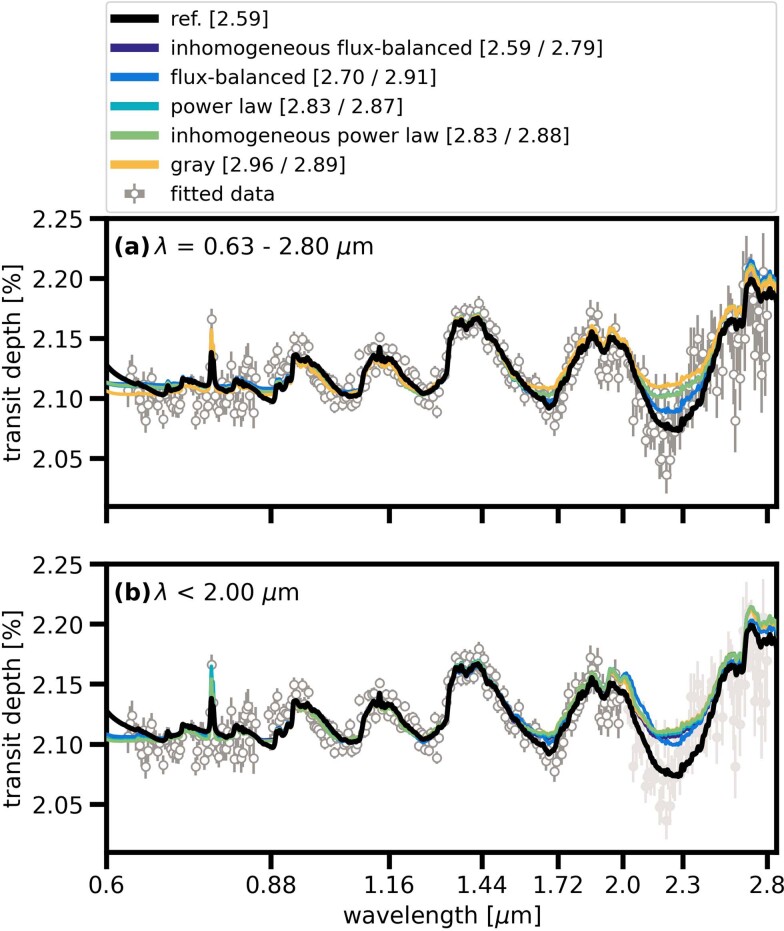
A demonstration of how the redder wavelength coverage of NIRISS/SOSS drives the inference on cloud structure for WASP-39b. We fit the NIRISS/SOSS spectrum (grey) using a suite of cloud models to derive the best-fit C/O ratio and metallicity. Here we demonstrate how the best-fit model for each cloud treatment changes as a function of what wavelength region we fit. **a**, The best-fit models when using the entire wavelength coverage of NIRISS/SOSS. **b**, The best-fit models when using *λ* < 2 μm, which excludes the overlapping region between orders on the detector. The reference spectrum (black) on both panels corresponds to the best-fit inhomogeneous droplet sedimentation model for the entire wavelength coverage. The fitted data are presented as dark-grey points. The quoted numbers in brackets in the legend are the respective *χ*^2^/*N* for each fit for the top (left value) and bottom (right value). The difference between cloud models is within the noise of the NIRISS/SOSS data when fitting to *λ* < 2 μm. It is clear that fitting the entire NIRISS/SOSS wavelength coverage results in a lower *χ*^2^/*N* and better fit. (https://github.com/afeinstein20/wasp39b_niriss_paper/blob/main/scripts/edfigure7.py).

The NIRISS/SOSS observations enable the detection of clouds in the atmosphere of WASP-39b. Clear atmosphere models cannot explain the amplitudes of all of the water-vapour features simultaneously, which strongly indicates the presence of clouds (see Methods and Extended Data Fig. 6). The atmospheric models explored here indicate the presence of non-grey and non-homogeneous clouds, with model preferences of 8*σ* and greater for models with both non-grey and non-homogeneous clouds over models with grey homogeneous clouds only. This model preference is driven by the decrease in transit depth between 2.0 and 2.3 μm (see Extended Data Fig. 7a), which cannot be explained by grey clouds uniformly distributed along the terminator (see Extended Data Fig. 7a). Moreover, in the various cloud treatments tested here (grey, grey + power law and flux-balanced clouds; see Methods), both parametric and droplet sedimentation models indicate a preference for inhomogeneous cloud coverage of roughly 50–70% around the planetary day–night terminator because it better explains the decrease in transit depth between 2.0 and 2.3 μm.

Atmospheric circulation and cloud microphysical models have predicted that the cloud structure varies substantially along the terminators of hot Jupiters^31–33^. In particular, different compositions of clouds have different condensation temperatures and thus probably have different cloud coverage at the terminator^31^. Further studies combining temperature difference of east–west terminators to microphysical cloud models may be able to use the measured cloud coverage to determine the cloud composition of WASP-39b. Previous indications of non-grey or non-homogeneous clouds^34–39^ have relied on a single or small number of spectroscopic points, making our inference here for WASP-39b of non-grey cloud with inhomogeneous terminator coverage in the transmission spectrum of an exoplanetary atmosphere the most confident so far. These constraints on the physical properties of clouds, alongside several spectral features across a broad wavelength coverage, are key to breaking well-known degeneracies between the metallicity and cloud cover in atmospheric models^8,14,40^ and deriving constraints on the bulk atmospheric properties.

The high precision of NIRISS/SOSS in combination with broadest wavelength coverage <2.8 μm for any JWST instrument, minimal systematics and no issue with saturation allows us to obtain more precise and robust constraints on atmospheric composition and tracers of planet formation than most previous transmission spectroscopy observations. The super-solar metallicity of WASP-39 b and the solar-to-super-solar K/O ratio are in agreement with previous studies of mass-metallicity trends in transiting exoplanets^3,7,27,41^. If confirmed with further detailed modelling, a super-solar K/O ratio in the atmosphere of WASP-39 b would probably indicate enrichment resulting from the accretion of planetesimals^25–27^, although the measurements of potassium and oxygen abundances for the host star are also needed to establish this result. Similarly, the suggestion of sub-solar C/O ratio and super-solar metallicity may be compatible with a planetesimal accretion scenario, for example, refs. ^42–44^. The combination of a super-solar metallicity, super-solar K/O ratio and sub-solar C/O ratio may suggest that the planet formed beyond the H_2_O snow line followed by inward migration, for which theory predicts efficient accretion of planetesimals at approximately 2–10 AU, for example, refs. ^25,45^. At those orbital distances, the planetesimals probably contain K rock (for example, alkali feldspar KAlSi_3_O_8_ (refs. ^46,47^)) and H_2_O ice but almost no CO ice, for example, refs. ^48,49^, which explains the sub-solar C/O ratio and super-solar K/O ratio, along with a super-solar metallicity if a sufficient amount of planetesimals was accreted. However, fully understanding the possible formation pathways of this planet requires statistical constraints on the complete chemical inventory of the planet and the relative abundances of the carbon-bearing, oxygen-bearing and alkali-bearing species. Such efforts will be possible when applying retrieval techniques to the complete transmission spectrum of WASP-39b from 0.5 to 5.5 μm that is being produced by the Transiting Exoplanet Community Early Release Science Program. Our results validate the JWST’s NIRISS/SOSS as an instrument mode fully capable of producing excellent exoplanet atmosphere measurements.

## Methods

Given the newness of the data, we applied six independent data-reduction and light-curve-fitting routines to the data: nirHiss, supreme-SPOON, transitspectroscopy, NAMELESS, iraclis and FIREFLy. Each pipeline extracts the stellar spectra from orders 1 and 2 (*λ* = 0.6–2.8 μm) with the exception of FIREFLy, which only extracts data from order 1. There is an extra order 3 that has a spectral range of *λ* = 0.6–0.95 μm (ref. ^50^). However, the signal of order 3 is generally weak and, because it provides no new wavelength information beyond what is covered in orders 1 and 2, is not used by any of the presented pipelines. Below, we first describe the important reduction steps taken by each, followed by their light-curve-fitting methodologies. We note here that, in each pipeline, the position of the SOSS trace was found to match almost perfectly with that measured during commissioning (Fig. 1). Furthermore, each pipeline trimmed the first 10–15 integrations to remove the effects of the exponential ramp in the fitting routines. We present a summary of all pipelines in Extended Data Table 1.

### The nirHiss pipeline

nirHiss is a Python open-source package that uses the stage 2 outputs from the Eureka! pipeline and performs further background and cosmic-ray removal, as well as extraction of the stellar spectra. For this analysis, we took the uncalibrated images and ran our own stages 1 and 2 calibration using Eureka!^51^, an open-source package that performs spectral extraction and light-curve fitting for several JWST instruments. We use the default steps presented in Eureka!, which includes detector-level corrections, production of count-rate images, application of physical corrections and calibrations to individual exposures.

Next, nirHiss removes background noise sources in a multistep process. The zodiacal background is first removed by applying the background model provided on the STScI JDox User Documentation website (https://jwst-docs.stsci.edu/). The background is scaled to a small region of each science integration in which there was no contamination from any of the orders; in this case, *x* ∈ [190, 250], *y* ∈ [200, 500]. The average scaling—calculated here to be 0.881—is applied to all science integrations. Second, a model of zeroth-order contaminants is built using the F277W integrations. The F277W integrations were taken after the transit of WASP-39b with the GR700XD/CLEAR pupil element and the F277W filter (throughput centred at *λ* = 2.776 μm with a bandwidth of *λ* = 0.715 μm). These observations consist of ten integrations with an exposure time of 49.4 s. Observations with the F277W filter contain only the spectral trace of order 1 in the region in which *x* ≤ 460 pixels, thus allowing for the detection and modelling of zeroth-order contaminants across most of the detector. A median F277W frame is created to identify and mask any bad-data-quality pixels.

To ensure that no further noise is added from the median F277W frame, we create a 2D background model map using photutils.Background2D. To identify regions of the background, we masked the upper-left corner, in which the trace is located, and any regions >1.5*σ*, which includes the zeroth-order sources. For photutils.Background2D, we used a filter size of (3, 2) pixels and a box size of (2, 2) pixels. Once the background is removed from the median F277W frame, we apply a Gaussian filter with a width of 2 to smooth out any further small-scale background noise. To apply the median F277W frame to the stage 2 science integrations, we scaled it to two isolated zeroth-order sources in the science integrations at *x*_1_ ∈ [900, 1,100], *y*_1_ ∈ [150, 250] and *x*_2_ ∈ [1,800, 2,000], *y*_2_ ∈ [150, 250]. We applied the average scaling to all integrations. We found the average F277W background scaling to be 2.81. We apply the scaled background frame to each time-series observation integration.

Once the zeroth-order contaminants are removed, we trace the location of orders 1 and 2. The spatial profile for NIRISS/SOSS along the column is double-peaked, with a slight dip in the middle. We developed a routine to identify the trace locations using a three-step approach to identifying each order. For each column in the first order trace, we identify the locations of the two peaks, or ‘ears’, and assume that the middle of the trace is the median row pixel between the two ears. We repeat this process for the third and second orders in that sequence, masking orders once they have been traced. We chose to identify the third order before the second order because it is better spatially resolved and does not overlap with any other orders. The routine creates one main set of traces from a median frame of all observations, which is used to extract the stellar spectra. As an extra output, we track the changes in the (*x*, *y*) pixel positions of each order on the detector across all integrations.

After the traces are identified, we continue our reduction to remove any extra noise and cosmic rays/bad pixels. We perform further 1/*f* noise correction following the routine presented in transitspectroscopy (described below). Finally, nirHiss identifies and interpolates over cosmic rays. To identify cosmic rays, we used the L.A.Cosmic technique wrapped into ccdproc^52,53^, which identifies pixels based on a variation of the Laplacian edge detection. We identify cosmic rays as pixels with *σ* > 4 using this method. We interpolate over any further bad pixels by taking the median value of the two surrounding pixels along the column. We extract the spectra using a box-extraction routine and ignore any contaminants from overlapping orders or from any potential background orders. We use a box diameter of 24 pixels for both orders 1 and 2.

### The supreme-SPOON pipeline

In parallel, we reduce the WASP-39 b time-series observation with the independent supreme-SPOON (supreme-Steps to Process sOss ObservatioNs) pipeline, which processes SOSS time-series observations from the raw, uncalibrated detector images to extracted 1D light curves. An outline of the specific steps is presented below.

For detector-level processing, supreme-SPOON closely follows stage 1 of the jwst pipeline. All default steps, up to and including the reference pixel correction, are run using their default settings. The reference pixel step is known to provide an inadequate correction of 1/*f* noise for SOSS observations; however, we include it to remove group-to-group variations in the bias level, as well as even–odd row variations. At this stage, we remove the zodiacal background from each group. This is accomplished by first calculating a group-wise median frame and scaling the model background provided in the STScI JDox to the flux level of each group in this median, yielding eight background models, one for each group. The region chosen to calculate the scaling was *x* ∈ [300, 500], *y* ∈ [210, 250], in which there is minimal contamination from any of the SOSS orders. The *n*th background model is then subtracted from the corresponding group of each integration.

We then proceed to a more in-depth treatment of 1/*f* noise. Unlike the other pipelines used in this work, supreme-SPOON treats 1/*f* noise at the group level instead of at the integration level. 1/*f* noise is a time-varying noise source introduced by the voltage amplifiers during the readout of the detector and therefore the 1/*f* pattern will vary from group to group, even within a given integration. To perform the 1/*f* correction, first a median out-of-transit frame is calculated for each group. This group-wise median is then scaled to the flux level of each frame in a given group by means of the transit curve and subtracted, showing the characteristic 1/*f* striping in the residuals. A column-wise median of this residual map is then subtracted from the original frame. The trace residuals as well as any bad pixels are masked in the median calculation.

From this point, we once again proceed with the standard stage 1 steps of the jwst pipeline, with the exception of the dark current step, to obtain the supreme-SPOON stage 1 outputs. The dark current subtraction step is skipped as it was found to reintroduce 1/*f* noise into the data. The dark current level is also extremely small (several tens of electrons s^−1^ compared with many thousands for the target signal) and can thus be safely ignored. supreme-SPOON only applies the assign_wcs, srctype and flat_field steps of the stage 2 jwst pipeline to the stage 1 products. The background subtraction was already performed as part of stage 1 calibrations. Furthermore, the flux calibration steps (pathloss, which accounts for light incident on the telescope primary mirror that falls outside the SUBSTRIP256 subarray, and photom, which performs the actual photometric flux calibration) are skipped, both because an absolute flux calibration is unnecessary for relative spectrophotometric measurements and a wavelength-dependent flux calibration is nonsensical for SOSS, in which contributions from several wavelengths from all orders affect a single pixel. At this point, supreme-SPOON identifies any remaining hot pixels through median filtering of a median stack of all frames and interpolates them by means of the median of a surrounding box. These products are the supreme-SPOON stage 2 results.

Stage 3 of the supreme-SPOON pipeline is the 1D extraction. This can be performed through two different methods: the first is a simple box aperture extraction on each order, ignoring the order contamination. The second uses ATOCA (Algorithm to Treat Order ContAmination)^50^ to explicitly model the order contamination. Briefly, ATOCA constructs a linear model for each pixel on the detector, including contributions from the first and second diffraction orders, allowing for the decontamination of the SOSS detector—that is, ATOCA constructs models of both the first and second orders individually, thereby allowing a box extraction to be performed on each free from the effects of order contamination. Although the effects of this order contamination for differential measurements (such as exoplanet atmosphere observations) are predicted to be small (about 1% of the amplitude of the expected spectral features)^50,54^, in the quest to obtain the most accurate possible transmission spectra, this contamination effect is important to take into account. ATOCA is at present built into the Extract1dStep of the jwst pipeline, although it is not the default option and must be toggled to on by means of the ‘soss_atoca’ parameter. To improve the performance of ATOCA, we do not use the default specprofile reference file included in the jwst pipeline but instead construct estimates of the underlying spatial profiles of the first and second orders, on which ATOCA relies, using the APPLESOSS (A Producer of ProfiLEs for SOSS) algorithm^54^. We determine the centroid positions for each order on a median stack using the ‘edgetrigger’ algorithm^54^, and these positions are found to match to within a pixel with the default centroids contained in the jwst_niriss_spectrace_0023.fits reference file; the spectrace file is available on the JWST Calibration Reference Data System (CRDS). The SOSS trace position is furthermore highly stable over the course of this time-series observation, with root mean square (RMS) variations in *x* and *y* positions of approximately 5 mpix and RMS rotation of about 0.3″. We therefore fix the ‘soss_transform’ parameter to [0, 0, 0] and perform the extraction with a box size of 25 pixels. Any remaining >5*σ* outliers in the resulting spectra are then identified and clipped. At present, supreme-SPOON does not explicitly treat contamination from zeroth orders of background stars that intersect the trace.

### The transitspectroscopy pipeline

This third pipeline analysis combines the jwst pipeline stage 1 ‘rateints.fits’ files with transitspectroscopy^55^. transitspectroscopy completes stellar spectral extraction as well as transit fitting.

The trace positions for NIRISS orders 1 and 2 were determined using transitspectroscopy.trace_spectrum. This routine cross-correlates an input function with each column in the detector to find the centre of the different traces by means of the maximum of the resulting cross-correlation function. To follow the shape of the NIRISS order profiles, an input function consisting of a double Gaussian was used with parameters that were trained on the NIRISS/SOSS observations of HAT-P-14 b (JWST Program ID 1541; principal investigator Espinoza): *μ*_1_ = −7.5; *σ*_1_ = 3.0; *μ*_2_ = 7.5; *σ*_2_ = 3.0. The trace for order 2 was not fit for pixels ≤1,040, as the throughput is not high enough for the method to robustly fit the trace without incorporating nearby contaminants. After identifying the trace positions with this method for both orders 1 and 2, both traces were smoothed using a series of spline functions. We find that the best-fit parameters for order 1, which were trained on the HAT-P-14 b observations, are: *x*_knots,1_ = [[6, 1,200-5], [1,200, 1,500-5], [1,500, 1,700-5], [1,700, 2,041]]; *n*_knots,1_ = [4, 2, 3, 4] and for order 2: *x*_knots,2_ = [[601, 850-5], [850, 1,100-5], [1,100, 1,749]]; *n*_knots,2_ = [2, 2, 5].

The zodiacal background was removed by scaling the model background provided on the STScI JDox User Documentation. This model was compared with a small region of the median science integrations in which there was little to no contamination from the orders (*x* ∈ [500, 800], *y* ∈ [210, 250]). The ratio of all the pixels in this region versus the pixels in the background model was computed, ordered and the median ratio of all the second quartile pixels was used as the scaling factor between the background model and the data, which was found to be 0.909. All the integrations had this scaled background subtracted.

Each integration is corrected for 1/*f* noise with the following procedure. First, all the out-of-transit, background-corrected integrations are median combined and scaled by the relative flux decrease produced by the transit event at each integration (that is, 1.0 for out-of-transit integrations or about 0.976 for mid-transit). These scaled median frames are then subtracted from each individual integration, which then leaves in the frame only detector-level effects, such as 1/*f* noise. We then go column by column and take the median of all pixels in these residual frames within a distance of 20–35 pixels from the centre of the trace, and use this as an estimate of the contribution from 1/*f* noise to that given column. This value is then removed from each pixel within 20 pixels from the trace on that column. No correction for order 1 contamination on order 2 was made, as the contribution is negligibly small in this case^50^—similarly for order 1 contamination in order 2 in our extraction.

To extract the resulting background-corrected and 1/*f*-corrected spectrum, we used the transitspectroscopy.spectroscopy.getSimpleSpectrum routine with a 30-pixel total aperture for both orders. To handle obvious outliers in the resulting spectrum because of, for example, uncorrected cosmic rays and/or deviating pixels, we median-normalized the spectra for each integration and combined them to form a ‘master’ 1D spectrum for both orders 1 and 2. The median was taken at each wavelength, as well as the error on that median, and this was then used to search for 5*σ* outliers on each individual integration at each wavelength. If outliers were found, they were replaced by the rescaled version of this median master spectrum.

### The NAMELESS pipeline

Starting from the jwst pipeline stage 1 products, we use the NAMELESS (Niriss dAta reduction MEthod for exopLanEt SpectroScopy) pipeline to go through the jwst pipeline stage 2 with the addition of custom correction routines.

First, we go through the assign_wcs, srctype and flat_field steps of the jwst pipeline stage 2, opting for a custom background-subtraction routine and skipping the pathloss and photom steps as absolute flux calibration is not needed. After flat-field correction, we scale the model background provided on the STScI JDox User Documentation to a region of the median frame in which the contribution from the tail of the three orders is lowest (*x* ∈ [200, 250], *y* ∈ [400, 600]). From the distribution of the scaling values of all pixels within the defined region, we take the 16th percentile as our scaling value and subtract the scaled background frame from all integrations.

We subsequently correct for 1/*f* noise by performing a column-by-column subtraction for each median-frame-subtracted integrations. The median frame is computed from the out-of-eclipse integrations (integration # ∈ [200, 400]) and scaled to each individual integration by dividing the sum of the pixels in the first order by that of the median frame. We then subtract the scaled median frame from all integrations, perform the column-by-column subtraction on the residual frames and add back the scaled median frame to the corrected residual frames to obtain the 1/*f*-corrected integrations.

We detect outliers frame by frame using the product of the second derivatives in the column and row directions. This method works particularly well for isolated outliers, as this leads to a strong inflexion that corresponds to a large second derivative. Because the spectral orders also lead to larger second-derivative values, we divide the frames into windows of 4 × 4 pixels, compute the local second-derivative median and standard deviation and flag any pixel that is more than four standard deviations away from the median. Furthermore, we also flag pixels with null or negative flux. All identified outliers are set equal to the median value of the window in which it was identified.

Finally, we proceed with spectral extraction of the corrected frames by first tracing the sections of the spectral orders that we wish to extract. We trace orders 1 and 2 from *x*_1_ ∈ [4, 2,043] and *x*_2_ ∈ [4, 1,830], respectively. The centre of the traces is found for each individual column by performing a convolution of the profile with a Gaussian filter, in which we use the maximum of the convolved profile as the centre of the trace. For the tracing of the second order, we keep the centre of the trace fixed below *x* = 900, as the flux from the first order can bias the tracing method. Furthermore, we smooth the positions of the trace centroids using a spline function with 11 and 7 knots for the first and second orders, respectively. We perform spectral extraction of the first and second orders at all integrations using the transitspectroscopy.spectroscopy.getSimpleSpectrum routine with an aperture width of 30 pixels.

### The iraclis pipeline

We used the jwst pipeline stage 1 rateints.fits files with modified routines from iraclis^5,56^, which was initially designed for the HST. The modified routines will be part of the version 2 of iraclis, which will become publicly available in the near future. The routines applied to the rateints.fits files were flat fielding, bad-pixels and cosmic-rays correction, sky background subtraction, 1/*f* noise correction, *X*-drift and *Y*-drift detection, light-curve extraction, light-curve modelling and planetary spectrum decontamination.

We started our analysis by dividing the images by the appropriate flat-field frame (jwst_niriss_flat_0275.fits), as provided by the JWST CRDS. The next step was the bad-pixels and cosmic-rays correction. For bad pixels, we used those with a positive DQ flag in the rateints.fits files, excluding the warm pixels, as their large number did not allow for a reliable correction. We also identified extra outliers (cosmic rays or other artefacts) by calculating two flags for each pixel: the difference from the average of the ten horizontally neighbouring pixels (*x*-flag) and the difference from the average of the ten vertically neighbouring pixels (*y*-flag). If a pixel’s *x*-flag was 5*σ* larger than the other pixels in the column and its *y*-flag 5*σ* was larger than the other pixels in the row, it was identified as a cosmic ray (see also ref. ^56^). Both bad pixels and outliers were replaced with the value of a 2D interpolation function, created from the rest of the pixels, similarly to analyses with the HST^56^.

We then subtracted a column-based sky background frame and a column-based 1/*f* noise frame from each image. For each image, we first used a trace filter (value >0.001 in the jwst_niriss_spectrace_0023.fits, provided by the JWST CRDS) and a column-based 1 × median absolute deviation filter to find the illuminated pixels. Then, we calculated the column-based median of the image—using only the unilluminated pixels—and subtracted it from the image. Finally, we calculated the column-based median of the IMFD (Image-MedianFrame Difference)—using only the unilluminated pixels—and subtracted it from the image. This process is not efficient in subtracting 100% of the background contamination, which was removed during the last analysis step (spectrum decontamination).

*X*-pixel and *Y*-pixel trace drifts were detected relative to the first image by comparing the sums along the columns and the rows, respectively, similarly to the HST^56^. The drifts are on the order of  pixels without any evident trend in motion. Because this is below the subpixel size used in the iraclis extraction, we find that there is no marked impact of not correcting these drifts. For each spectroscopic image, we initially divided each pixel into a 100 × 100 grid of subpixels and, for each subpixel, we calculated the distance from the trace (CD) and the wavelength (*λ*), creating the CD_map_ and the *λ*_map_, respectively. *λ* was assigned to each subpixel directly from the wavelength solution (interpolated wavelength solution from the jwst_niriss_wavemap_0013.fits file, provided by the JWST CRDS, shifted by the detected *X* and *Y* drifts). CD was calculated as the distance between the centre of the subpixel and the point of the trace with the same distance (interpolated trace from the jwst_niriss_spectrace_0023.fits file, provided by the JWST CRDS, shifted by the detected *X* and *Y* drifts). Our high-resolution bins had a *λ* width of 10 Å, ranging between 0.62 and 0.85 μm for order 2 and between 0.85 and 2.8 μm for order 1, and a CD width of 1.5 pixels, ranging from −25 to 25 pixels.

Finally, to construct the light curve of each bin, we applied the following smoothed aperture mask on each spectroscopic image and summed the values of all the subpixels. We chose a smoothed aperture, similarly to the HST to reduce the effects of jitter noise:

$${\rm{MASK}}=0.5\times \left[{\rm{erf}}\left(\frac{\left({{\rm{CD}}}_{{\rm{map}}}-{{\rm{CD}}}_{1}\right)}{{\sigma }_{{\rm{CD}}}\sqrt{2}}\right)-{\rm{erf}}\left(\frac{\left({{\rm{CD}}}_{{\rm{map}}}-{{\rm{CD}}}_{2}\right)}{{\sigma }_{{\rm{CD}}}\sqrt{2}}\right)\right]\times \left[{\rm{erf}}\left(\frac{\left({\lambda }_{{\rm{map}}}-{\lambda }_{1}\right)}{{\sigma }_{\lambda }\sqrt{2}}\right)-{\rm{erf}}\left(\frac{\left({\lambda }_{{\rm{map}}}-{\lambda }_{2}\right)}{{\sigma }_{\lambda }\sqrt{2}}\right)\right]$$

in which CD_1_, CD_2_ and *σ*_CD_ are the bin boundaries and the smoothing factor along the cross-dispersion axis, respectively, and *λ*_1_, *λ*_2_ and *σ*_*λ*_ are the bin boundaries and the smoothing factor along the dispersion axis, respectively. For the smoothing factors, we used the values of *σ*_CD_ = 0.015  pixels and  Å —that is, about 10% of the bin size. We chose these values for the smoothing factors because lower values would effectively create a sharp-edge aperture, whereas larger values would force the bins to overlap substantially.

### FIREFLy

Although FIREFLy (Fast InfraRed Exoplanet Fitting for Light curves)^57^ was written and optimized for reducing NIRSpec-PRISM and G395H time-series observations, it worked well on the NIRISS/SOSS dataset, in which it selected and processed the spectrophotometry from order 1 only with minimal tuning or intervention. FIREFLy is not written in such a way to extract data from order 2 (*λ* < 0.9 μm). In our reduction, we perform standard calibrations on the raw data using the jwst pipeline for stages 1 and 2 reduction. On the jwst stage 2 outputs, we perform bad-pixel and cosmic-ray cleaning on each integration. We perform 1/*f* destriping and background subtraction using a pixel mask generated from the temporally medianed image that selects regions of the data image below a specified count threshold. We extract the spectrophotometry using an optimized aperture extraction width that is constant in wavelength. The aperture width is selected such that the scatter of the resulting out-of-transit white-light photometry is minimized.

### Light-curve fitting and transmission spectra

**Extended Data Fig. 8:**
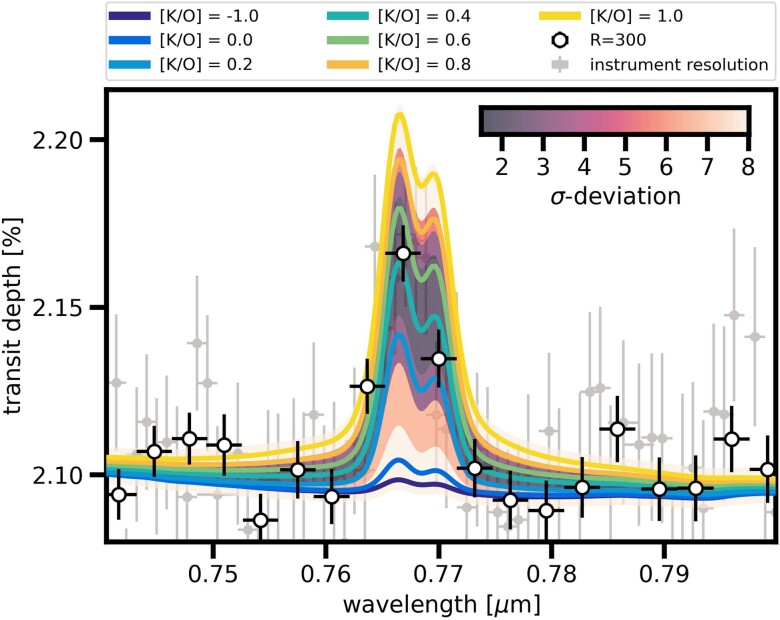
Evidence for super-solar K/O ratio in WASP-39b. We fit for the K/O ratio while keeping the rest of the model parameters (for example, C/O ratio, metallicity and redistribution) the same as our reference model and fitting for the cloud parameters and scaled planetary radius. Here we present the different K/O ratio models (solid lines) we fit against the transmission spectrum at *R* = 300 (black and white points). We represent the respective fit of each model in the orange shading. (https://github.com/afeinstein20/wasp39b_niriss_paper/blob/main/scripts/edfigure8.py).

We used a suite of light-curve-fitting routines to fit the extracted light curves. Each routine fits for orbital parameters from the broadband white-light curves for each order (see Extended Data Fig. 8). We fixed the orbital period to the best-fitting value from *P* = 4.05528 days (ref. ^1^) for all pipeline fits. For the spectroscopic light curves, most routines (nirHiss/chromatic_fitting, supreme-SPOON/juliet, transitspectroscopy/juliet and NAMELESS/ExoTEP) fixed the orbital parameters (that is, the mid-transit time, *t*_0_, semi-major axis to stellar radius ratio *a*/R_*_, impact parameter *b*, eccentricity *e*) to the same values to ensure consistency. These parameters were fixed to their best-fitting values from the transitspectroscopy/juliet white-light-curve fit, except for *t*_0_ which was fixed to the value obtained from the white-light curve in each case. This left the planet-to-star radius ratio *R*_p_/R_*_, the limb-darkening coefficients and parameters for any further systematics models to vary. These four routines also fit spectroscopic light curves at the native instrument resolution. However, two routines, iraclis and FIREFLy, instead fixed the orbital parameters in their spectroscopic fits to values obtained through their white-light curve fits. iraclis also fits directly for the orbital inclination, *i*, as opposed to *b* and *a*/R_*_ like the other routines. iraclis fits for their spectrophotometric light curves at the pixel resolution, whereas FIREFLy binned the spectroscopic light curves first and fits for the transit parameters. We present all of the best-fit white-light-curve parameters for order 1 in Extended Data Table 2. Furthermore, for the spectroscopic light-curve fits, we only considered the region of order 2 with wavelength <0.85 µm, as the 0.85–1.0-µm range is covered at higher signal-to-noise ratio (SNR) by order 1. All errors on each parameter are representative of 1*σ* (lower 16th and upper 84th percentiles) of the fit.

### chromatic_fitting

chromatic_fitting is an open-source Python tool for modelling multi-wavelength photometric light curves. This tool is built on the framework of chromatic, a package for importing, visualizing and comparing spectroscopic datasets from a variety of sources, including Eureka! and the jwst pipeline. In this paper, we applied chromatic_fitting to the nirHiss reduction.

chromatic_fitting uses the PyMC3 (NUTS) sampler^58^ to fit the exoplanet transit model to the light curves. First, we fit the white-light curves for order 1. The white-light curve was generated using an inverse variance-weighted average of the unbinned data. We fixed the orbital period to 4.05528 days (ref. ^1^) and assumed a circular orbit. We fit for the mid-transit epoch *t*_0_, the stellar mass M_*_ and radius R_*_, the impact parameter *b*, the planet-to-star radius ratio *R*_p_/R_*_, quadratic limb-darkening coefficients (*u*_1_, *u*_2_) and out-of-transit flux *F*_0_. For the fitted parameters *t*_0_, M_*_, R_*_, *R*_p_/R_*_ and *F*_0_, we assumed normal priors N(2459787.56, 0.02^2^), N(0.934, 0.056^2^), N(0.932, 0.014^2^), N(0.146, 0.05^2^) and N(1.0, 0.01^2^), respectively. For *b*, we used a uniform prior between 0 and 1.146, in which *b* ≤ 1 + *R*_p_/R_*_. For the limb-darkening coefficients, we calculated the theoretical values from 3D models in ExoTIC-LD^59–61^ (based on the stellar parameters *T*_eff_ = 5,512 K, log*g* *=* 4.47 dex and Fe/H = 0.0 dex (ref. ^17^)) and assumed normal priors around these values with *σ* = 0.05. We also included a second-order polynomial in time to describe the systematics with a fixed constant term of 0.0 and normal priors on the first-order and second-order coefficients *c*_1_ and *c*_2_ of N(0.0, (1*e*^−4^)^2^). Using the NUTS implementation of PyMC3, we ran 4,000 tuning steps and 4,000 draws, with four walkers, for the white-light curve and the mean parameter values are shown in Extended Data Table 2. We checked for convergence using the rank-normalized R-hat diagnostic^62,63^.

For each spectroscopic light curve, we fixed the period *P*, transit epoch *t*_0_, eccentricity *e*, semi-major axis in stellar radii *a*/R_*_ and impact parameter *b* to the white-light parameter values from the transitspectroscopy/juliet routine (Extended Data Table 2). We then fit for the planet-to-star radius ratio *R*_p_/R_*_, quadratic limb-darkening coefficients (*u*_1_, *u*_2_) and out-of-transit flux *F*_0_—for all of these parameters, we assumed the same normal priors as for the white-light curve. We also included a second-order polynomial in time with the same priors as the white-light-curve fit. For each wavelength, we ran 2,000 tuning steps and 2,000 draws, also with four walkers. The final transmission spectrum was taken as the mean value drawn from the posterior distribution for the planet-to-star radius ratio with 1*σ* uncertainties extracted from the 16th to 84th highest density interval region.

The SNR in the spectrophotometric light curves from nirHiss for order 1 at 1.34 μm is 165 and that for order 2 at 0.71 μm is 103. We define the SNR as $\sqrt{{n}_{{\rm{bins}}}}\times {\sigma }_{{\rm{intransit}}}/{\sigma }_{{\rm{outoftransit}}}$, in which *σ* is the standard deviation.

### juliet

We applied the juliet package^64^ for light-curve fitting to the products of several reduction pipelines described above. Here we give a general overview of the methods and include exact details for each fit when appropriate.

For the supreme-SPOON reduced stellar spectra, we fit the white-light curve for the mid-transit time, *t*_0_, the impact parameter, *b*, the scaled orbital semi-major axis, *a*/R_*_ and the scaled planetary radius, *R*_p_/R_*_, assuming a circular orbit. We also fit two parameters of a quadratic limb-darkening model following the parameterization of ref. ^65^, as well as an additive scalar jitter and the two parameters of a linear trend with time. We therefore fit seven parameters to the white-light curve for each order, using wide, flat priors for each case. We then proceeded to fit the light curves from each individual wavelength bin at the native detector resolution—that is, two pixels per bin to roughly account for the extent of the point spread function in the spectral direction. This results in 1,020 bins for order 1 and 520 bins for order 2, as we only consider wavelengths <0.85 µm. For the spectroscopic fits, we fixed the central transit time to the white-light-curve value, and the other orbital parameters were as described for chromatic_fitting. For the linear trend with time, we used the white-light posterior for each of the two parameters as prior distributions for all wavelength bins, whereas for the limb-darkening parameters, we adopted a Gaussian prior centred around the predictions of the ExoTiC-LD package^61,66^ with a width of 0.1. As the SOSS throughput files included with ExoTiC-LD did not cover the full wavelength range of both orders, we instead used the throughputs determined during commissioning and included in the spectrace reference file of the jwst pipeline. We truncated the Gaussian prior at 0 and 1, to prevent the limb-darkening parameters from straying into unphysical regions of the parameter space. We then used flat, uninformative priors for the remaining two parameters, the scaled planetary radius and the scalar jitter. The supreme-SPOON white-light-curve fits have ${\chi }_{\nu }^{2}=1.15$ for order 1 and ${\chi }_{\nu }^{2}=1.11$ for order 2.

For the transitspectroscopy reduced stellar spectra, we first fit the white-light curves of orders 1 and 2 separately. For these, as suggested above, the period was fixed but the other parameters were allowed to vary. In particular, we set a normal prior on the time-of-transit centre of N(2459787.5, 0.2^2^) days, in which the first value denotes the mean and the second the variance of the prior. A normal prior was also set on *a*/R_*_ ~ *N*(11.37,0.5^2^), in which ‘~’ denotes ‘distributed as’, and a truncated normal between 0 and 1 was set for the impact parameter *b* ~ TN(0.447, 0.1^2^), in which the means were set following the work of ref. ^67^ but the variances are large to account for the variation of these parameters in the literature between different authors. We set a uniform prior for the planet-to-star radius ratio between 0 and 0.2 and fixed eccentricity to 0. As well as those, we fit for a mean out-of-transit offset with a normal prior of *N*(0, 0.1^2^) and a jitter term added in quadrature to the error bars with a log-uniform prior between 10 and 1,000 ppm. To account for systematic trends in the data, we use a Gaussian process by means of celerite^68^ with a simple Matérn 3/2 kernel to parameterize those trends. We set log-uniform priors for both the amplitude of this Gaussian process from 10^−5^ to 1,000 ppm and for the timescale from 10^−3^ days to 0.5 days. We use the framework of ref. ^65^ to parameterize limb darkening through a square-root law, which, following ref. ^69^, is one of the laws that should give the best results at this level of precision.

For the wavelength-dependent light curves, we used a similar setup with two main differences. The first is that we fix the time-of-transit centre, *a*/R_*_ and *b* to their white-light values. The second is that we set truncated normal priors on the transformed limb-darkening coefficients (*q*_1_, *q*_2_) between 0 and 1, with standard deviations of 0.1 and means obtained by the following method. First, we obtain the nonlinear limb-darkening coefficients using an ATLAS stellar model with the closest properties to those of WASP-39 using the limb-darkening software^70^. Then, the square-root law limb-darkening coefficients are obtained following the algorithm of ref. ^71^, which are transformed to the (*q*_1_, *q*_2_) parameterization using the equations in ref. ^65^. These are then set as the mean for each wavelength-dependent light curve. We note that we fit the light curves at the pixel level, which means fitting one light curve per detector column. We fit them in parallel using the transitspectroscopy.transitfitting.fit_lightcurves routine.

### ExoTEP

For the NAMELESS reduction, we perform light-curve fitting on the extracted spectrophotometric observations using the ExoTEP framework^72^. We first fit the white-light curves of both orders 1 and 2 separately. We fit for the mid-transit time *t*_0_, the planet-to-star radius ratio *R*_p_/R_*_ and quadratic limb-darkening coefficients (*u*_1_, *u*_2_)^73,74^, while fixing the impact parameter *b* and semi-major axis *a*/R_*_ to the values of the best order 1 white-light-curve fit from the transitspectroscopy/juliet analysis. We also fit for the scatter *σ*, as well as a linear systematics model with an offset *c* and slope *v*. Uniform priors are considered for all parameters. Furthermore, we only discard the first 10 min of observations (ten integrations) to remove the exponential ramp. For all light curves, we compute the rolling median for a window size of 11 integrations and bring any data point that is more than four standard deviations away from it to the median value. We fit the light curves using the Markov chain Monte Carlo (MCMC) ensemble sampler emcee^75^ for 1,000 steps using four walkers per free parameter. The first 600 steps, 60% of the total amount, are discarded as burn-in. We then fit the spectroscopic light curves, keeping *t*_0_ fixed to its white-light value, at a resolution of three pixels per bin for order 1 (680 bins) and one pixel per bin for order 2 from about 0.6–0.9 μm (675 bins). We used 1,000 steps for the spectroscopic fits, once again discarding the first 600 as burn-in.

### iraclis

We analysed all the light curves using the open-source Python package PyLightcurve^76^. For every light curve, PyLightcurve: (1) calculates the limb-darkening coefficients using the ExoTETHyS package^77,78^, the wavelength range of the bin, the response curves for each of the NIRISS orders (jwst_niriss_spectrace_0023.fits file, provided by the JWST CRDS) and the stellar parameters (*T*_eff_ = 5,540 K, log*g* = 4.42 cm s^−2^, Fe/H = 0.14 dex (ref. ^79^)); (2) finds the maximum-likelihood model for the data (an exposure-integrated transit model together with a quadratic trend model using the Nelder–Mead minimization algorithm included in the SciPy package^80^; (3) removes outliers that deviate from the maximum-likelihood model by more than three times the standard deviation of the normalized residuals; (4) scales the uncertainties by the RMS of the normalized residuals, to take into account any extra scatter; (5) and, finally, performs an MCMC optimization process using the emcee package^75^. We initially modelled the first-order white-light curve (sum of all bins above 0.85 μm with out-of-transit fluxes above 20 data numbers per second (DN s^−1^) and fit for the white *R*_p_/R_*_, the orbital parameters, *a*/R_*_ and *i*, and the transit mid-time. We then modelled the spectral light curves, fitting only for the *R*_p_/R_*_, fixing the orbital parameters, *a*/R_*_ and *i*, and the transit mid-time to the above white results. In both cases, the models also included a quadratic detrending function that was multiplied by the transit model. After modelling, we applied a spectral decontamination step, taking advantage of the varying total flux across the spectral traces. Owing to the contamination, we have (*R*_p_/R_*_)^2^ × (TF − *x*)/TF, in which TF is the out-of-transit flux (star and contamination) and *x* is the flux of the contaminating source. Hence, for each wavelength, we fitted for *x* and applied the correction ${({R}_{{\rm{p}}}/{R}_{* })}_{{\rm{corr}}}^{2}={({R}_{{\rm{p}}}/{R}_{* })}^{2}\times {\rm{TF}}/({\rm{TF}}-x)$. This procedure is effective in removing uniform contamination. The uniform contamination fixes issues of sky background overcorrection or undercorrection. It also corrects for order overlap. After the decontamination described above, there was still a contaminating source affecting the spectrum around 0.72 μm, which was not uniform because of the point spread function. To separate this source, we applied independent component analysis on the stellar spectra extracted from various distances from the trace. We used two components to describe the contaminating source and one to describe the stellar spectrum. Finally, we estimated the (*R*_p_/R_*_)^2^ for each wavelength bin using the weighted average of all the bins that had the same wavelength range. We only took into account the bins that had out-of-transit fluxes above 20 DN s^−1^. This choice effectively applied an optimal aperture size for each wavelength bin.

### FIREFLy

To extract the transmission spectrum, we bin the cleaned spectrophotometric light curves by wavelength first to create 120 variable-width spectral channels with roughly equal counts in each. We fit for the transit depth of the planet in each channel using a joint light curve and systematics model. The systematics model accounts for spectral shifts in the *X* and *Y* directions^57^. We use the orbital parameters recovered from an MCMC fit to the white-light curve and fix them at each wavelength channel for our fit. We fit for the two quadratic limb-darkening terms *a* and *b* at each wavelength channel. We find that the best-fit limb-darkening coefficients are uniquely determined and deviate by a constant offset relative to model coefficients. Our fits are performed iteratively using the Python package lmfit. The light curves show a typical photometric scatter of 0.3% per integration and the typical transit-depth uncertainties vary between 150 and 300 ppm, which is in line with near-photon-limited precision. More details of the FIREFLy fitting routine can be found in ref. ^57^ and in ref. ^23^.

### Atmospheric models

To interpret the measured transmission spectrum, we performed an extensive comparison with grids of synthetic transmission spectra. We tested several independent atmospheric models to avoid any model-dependent interpretation of the data. Unless otherwise noted, all of our grids have assumed radiative–convective–thermochemical equilibrium to estimate atmospheric compositions. The exploration of atmospheric models with fewer assumptions (for example, without the assumption of chemical equilibrium with metallicity and C/O ratio as free parameters) and those considering other effects of disequilibrium chemistry is left for future work.

We derive basic interpretations for the observed spectrum based on four independent model grids, ATMO, PHOENIX, PICASO and ScCHIMERA. Each grid contains precomputed transmission spectra at various atmospheric properties, such as M/H, C/O ratio and cloud properties, using from grey to Mie-scattering cloud opacity (see next subsection for details). The ScCHIMERA grid considers further model advancements: (1) various cloud treatments, including grey cloud, grey + power-law cloud opacity and physically motivated (that is, droplet sedimentation) cloud model, (2) the impact of inhomogeneous cloud coverage along the planetary terminator and (3) K/O ratio as a grid dimension. ScCHIMERA provides the best fit to the observations compared with the other three grids and informs the results presented in the main text.

### Grid search with precomputed forward models

Here we introduce the independent grids of precomputed transmission spectra, their model description and the main results from these grid fits. We first present the three grids that assume horizontally homogeneous clouds.

### ATMO

The atmospheric pressure–temperature (PT) profile is computed using the 1D radiative–convective equilibrium model ATMO^81–83^. The model includes the molecular/atomic opacity of CH_4_, CO, CO_2_, C_2_H_2_, Cs, FeH, HCN, H_2_O, H_2_S, K, Li, Na, NH_3_, PH_3_, Rb, SO_2_, TiO and VO, for which the adopted line list is summarized in ref. ^84^. The line lists of several key species are: H_2_O (ref. ^85^), CH_4_ (ref. ^86^), CO_2_ (ref. ^87^), CO from HITEMP2010 (ref. ^88^) and K from VALD3 (ref. ^89^). We considered atmospheric metallicities M/H = −1.0, +0.0, +1.0, +1.7, +2.0 and +2.3, C/O ratios 0.35, 0.55, 0.7, 0.75, 1.0 and 1.5, planetary intrinsic temperatures *T*_int_ = 100, 200, 300 and 400 K and day–night energy redistribution factors of 0.25, 0.50, 0.75 and 1.00, for which full heat redistribution corresponds to 0.5. The model varies the O/H ratio to achieve each C/O ratio with a fixed C/H ratio, which is fixed then scaled to solar metallicity. The cloudy models include small particle opacity as the Rayleigh scattering gas opacity enhanced by a factor of either 0 or 10, whereas large particle opacity is equated to the H_2_ Rayleigh scattering opacity at 0.35 μm enhanced by a factor of 0.5, 1.0, 5.0, 10.0, 30.0 and 50.0. In total, the ATMO grid consists of 484 cloud-free and 6,292 cloudy atmosphere models. We only consider horizontally homogeneous clouds in the ATMO grid fits.

### PHOENIX

The atmospheric PT profile is computed using the 1D radiative–convective equilibrium model PHOENIX^90–92^. We considered atmospheric metallicities of M/H = −1.0, +0.0, +1.0 and +2.0, C/O ratio ranging from 0.3 to 1.0 divided into 136 grid points, planetary intrinsic temperatures *T*_int_ = 200 and 400 K and day–night energy redistribution factors of 0.172, 0.25 and 0.351, in which full heat redistribution corresponds to 0.25. The model varies the C/H ratio to achieve each C/O ratio with a fixed O/H ratio, which is also scaled to solar metallicity. The model includes various chemical species: CH, CH_4_, CN, CO, CO_2_, COF, C_2_, C_2_H_2_, C_2_H_4_, C_2_H_6_, CaH, CrH, FeH, HCN, HCl, HF, HI, HDO, HO_2_, H_2,_ H_2_S, H_2_O, H_2_O_2,_ H_3_^+^, MgH, NH, NH_3_, NO, N_2_, N_2_O, OH, O_2_, O_3_, PH_3_, SF_6_, SiH, SiO, SiO_2_, TiH, TiO, VO and atoms up to U. The line list of H_2_O is from BT2 (ref. ^85^), other molecular lines from HITRAN 2008 (ref. ^93^) and atomic lines from the database of Kurucz and Bell^94^. For cloudy models, the small non-grey cloud particle opacity is treated as a sum of Rayleigh scattering opacity of all gas species enhanced by a factor of either 0 (clear atmosphere) or 10; large grey particle opacity is treated as grey cloud deck pressure levels of 0.3, 3.0 and 10.0 mbar. In total, the PHOENIX grid consists of 95 cloud-free and 380 cloudy atmosphere models. We only consider horizontally homogeneous clouds in the PHOENIX grid fits.

### PICASO 3.0

Similarly to the grids of models presented above, we precomputed atmospheric PT profiles using the 1D radiative–convective equilibrium model PICASO 3.0 (refs. ^95–98^) for atmospheric metallicities M/H = −1.0, −0.5, +0.0, +0.5, +1.0, +1.5, +1.7 and +2.0, atmospheric bulk C/O ratios 0.229, 0.458, 0.687 and 1.100, planetary intrinsic temperatures *T*_int_ = 100, 200 and 300 K and heat redistribution factors of 0.5 and 0.4, in which full heat redistribution corresponds to 0.5. The model fixes the sum of C and O abundances (for example, the (C+O)/H ratio) to that scaled by the metallicity and solar C+O abundance. The model includes 29 chemical species: CH_4_, CO, CO_2_, C_2_H_2_, C_2_H_4_, C_2_H_6_, CrH, Cs, Fe, FeH, HCN, H_2_, H_2_O, H_2_S, H_3_^+^, OCS, K, Li, LiCl, LiH, MgH, NH_3_, N_2_, Na, PH_3_, Rb, SiO, TiO and VO. The line lists of several key species are: H_2_O (ref. ^99^), CH_4_ (ref. ^100^), CO_2_ (ref. ^101^), CO (ref. ^102^) and K from VALD3 (ref. ^89^). For cloudy models, we post-processed the computed PT profiles using the droplet sedimentation model Virga^15,103^, which determines the vertical distributions of cloud-mass mixing ratio and mean particle size from the balance between downward mass flux of gravitational sedimentation and upward mass flux of eddy diffusion. We vary vertically constant eddy diffusion coefficients of *K*_*zz*_ = 10^5^, 10^7^, 10^9^ and 10^11^ and vertically constant sedimentation parameters of *f*_sed_ = 0.6, 1.0, 3.0, 6.0 and 10.0. The *f*_sed_ value is defined as the ratio of the mass-averaged sedimentation velocity of cloud particles to the mean upward velocity of the atmosphere, with a smaller *f*_sed_ yielding more vertically extended clouds^99^; see, for example, refs. ^103,104^. We have assumed horizontally homogeneous clouds and accounted for the formation of MgSiO_3_, MnS and Na_2_S clouds. Then, we post-processed the atmospheric properties to compute synthetic transmission spectra. We note that the optical properties of the flux-balanced cloud are computed by the Mie theory^105^ under the assumption of a log-normal particle-size distribution with a mean particle size translated from *f*_sed_ (ref. ^15^). In total, the PICASO grid consists of 192 cloud-free and 3,840 cloudy atmosphere models.

We compare the NIRISS/SOSS spectrum (binned to *R* = 300) to each of these model grids and summarize the best fits in the top panel of Extended Data Fig. 6. For each cloudy and clear model we tested, we compute *χ*^2^/*N*_obs_ = 2.98–8.55 between the data and the models, with specific values per model indicated in the legend of Extended Data Fig. 6. All of our forward model grids consistently indicate super-solar metallicity (M/H = 1–2) and sub-solar C/O ratio. Each best-fit spectrum shows different structures at >2 μm, as the spectra at these wavelengths are more sensitive to the treatment of cloud properties (see next subsection for details). The best-fit spectra from PICASO, ATMO and PHOENIX indicate atmospheric metallicities of M/H = 1.7, 1.0 and 2.0, respectively. These models also consistently indicate that the C/O ratio is between 0.229 and 0.389, corresponding to the lowest C/O ratio grid point in each grid (see the main text for why models prefer lower C/O ratios). Thus, the super-solar metallicity and sub-solar C/O ratio of WASP-39b are consistent across the different model interpretations of the NIRISS/SOSS transmission spectrum.

We also find that clear atmospheric models fail to fit the observed spectrum even at very high metallicity (M/H = 2.0), as shown in the bottom panel of Extended Data Fig. 6. The clear models fail to match the amplitudes of H_2_O absorption features at *λ* = 0.90, 1.15, 1.40 and 1.80 μm simultaneously. The clear ATMO models fit the data better than the clear PICASO and PHOENIX models because the ATMO grid allows lower heat redistribution factors (that is, cooler atmosphere). The clear models also overestimate the transit depth at *λ* ≈ 2 μm because of a strong CO_2_ absorption resulting from the inferred high metallicity (M/H = 2.0). The inability of clear atmosphere models to fit the overall NIRISS spectrum strongly indicates the presence of clouds in the atmosphere and emphasizes the ability of the NIRISS wavelength coverage to break the cloud property–metallicity degeneracy. The best-fit cloud properties are *f*_sed_ = 1 and *K*_*zz*_ = 10^9^ cm^2^ s^−1^ for Virga clouds in PICASO, a grey cloud opacity of five times the H_2_ Rayleigh scattering opacity at 0.35 μm for ATMO and a grey cloud top pressure of 3 × 10^−4^ bar for PHOENIX.

### Grid search with ScCHIMERA

The NIRISS transmission spectrum offers key insights into the atmospheric properties of WASP-39b over a broad wavelength range. The simultaneous detection of H_2_O and K, alongside possible indications of carbon-bearing species, allows us to explore equilibrium models for which the K/O ratio is an extra dimension besides the commonly used C/O ratio and metallicity parameters. Furthermore, as explained in the previous subsection (see also Fig. 3 demonstrating how clouds contribute to the NIRISS spectrum), the broad wavelength coverage of these NIRISS observations makes it possible to explore more complex cloud models beyond traditional grey and homogeneous cloud models. To explore these considerations, we implement the ScCHIMERA grid as explained below.

### ScCHIMERA

Previous implementations of this framework include refs. ^106–108^, in which the methods are described in detail. Implementations of this procedure to the JWST data include ref. ^109^. For a given set of planetary parameters, our methods precompute the temperature–pressure structure of the planetary atmosphere and the thermochemical equilibrium gas-mixing-ratio profiles. The computations are performed on a grid of atmospheric metallicity (M/H, for example, log_10_ enrichment relative to solar^20^) spaced at 0.125 dex values between 0 and 2.25 (for example, 1–177 times solar) and C/O ratios at values of 0.20, 0.35, 0.45, 0.55, 0.65, 0.70 and 0.80. Unlike previous implementations of this framework, and to better understand the NIRISS/SOSS observations presented, we include a dimension to our grid exploring the K/O ratio (that is, log_10_ enrichment relative to solar^20^) with spacing of 0.5 dex between −1 and 0 and 0.1 dex between 0 and 1, overall spanning a range from −1 to 1 or 0.1–10 times solar. In these calculations, the atmospheric metallicity scales the sum of K, C and O. This sum determines the final elemental abundances after scaling metallicity, C/O ratio and K/O ratio. That is, the total oxygen elemental abundance is ${{\rm{O}}}^{{\prime} }=\frac{{\rm{M/H}}}{{\rm{K/O\; +\; C/O\; +\; 1}}}$, the total carbon elemental abundance is C′ = O′ × C/O and the total potassium elemental abundance is K′ = O′ × K/O. Furthermore, we explore the energy redistribution (*f*) between the day and night sides of the planet^110^, with values of 0.657, 0.721, 0.791, 0.865, 1.000, 1.030, 1.120, 1.217 and 1.319 in our grid, for which *f* = 1.0 and 2.0 correspond to full day-to-night heat redistribution and dayside-only redistribution, respectively.

The transmission spectrum of the planet is computed with CHIMERA^104,111–115^ using the converged atmospheric structures. We compare the observations to these models in a Bayesian inference framework using the nested sampling algorithm MultiNest^108^ through its Python implementation PyMultiNest^109^ and obtain an optimal set of M/H, C/O ratio, K/O ratio and *f* through nearest-neighbour search in the grid. When computing the transmission spectrum for a given set of (M/H, C/O ratio, K/O ratio, *f*), we also adjust the 1-bar planetary radius controlling the absolute transit depth (an arbitrary pressure with no direct impact on the inferred properties; see, for example, ref. ^8^) and model different cloud treatments. The opacity sources considered are H_2_–H_2_ and H_2_–He CIA^116^, H_2_O (refs. ^99,117^), CO_2_ (ref. ^117^), CO (ref. ^88^), CH_4_ (ref. ^88^), H_2_S (ref. ^118^), HCN (ref. ^119^), Na (refs. ^120,121^) and K (refs. ^120,122^), which were computed following the methods described in refs. ^123,124^. The cloud models considered are: (1) a basic cloud model with a grey, uniformly vertically distributed cloud opacity (*κ*_cloud_); (2) a grey + power-law cloud model that accounts for non-grey opacity of small-size particles as a vertically uniform power-law opacity (that is, a parameter for the scattering slope and a Rayleigh enhancement factor; for example, refs. ^13,36,39,125^) in addition to grey cloud component, which is expressed by a grey cloud deck of infinite opacity at a given atmospheric pressure; and (3) a droplet sedimentation model^15^ (assuming enstatite grains) in which parameters capture the eddy diffusion coefficient and the ratio of sedimentation velocity to characteristic vertical mixing velocity (see also the description of PICASO above). For cloud treatments 2 and 3, we also consider the possibility of inhomogeneities around the planetary limb by considering a linear combination of clear and cloudy models^14^, which is key for breaking degeneracies between metallicity and cloud properties^8,40^. We assume the same PT profile for both cloudy and clear limbs in the inhomogeneous cloud models and leave investigation on the possibility of different PT profiles in those regions to future studies.

### Identification of absorbers and model selection

We perform our Bayesian inference using all model combinations with the ScCHIMERA grid on four different data resolutions for the nirHiss transmission spectrum: *R* = 100, *R* = 300, native instrument resolution (*R*_order 1_ = 910; *R*_order 2_ = 830) and pixel-level resolution (*R*_order 1_ = 1,820; *R*_order 2_ = 1,660). Resolutions are given at the reference wavelengths of *λ* = 1.791 μm for order 1 and 0.744 μm for order 2. We test the robustness of our inferences against different binning and convolution strategies and find the results, that is, the bulk atmospheric properties M/H, C/O ratio and K/O ratio, to be consistent regardless of the resolution of the data. We find a fiducial combination of parameters that can best explain the spectrum (that we call the reference model) with full redistribution (*f* = 1, matching predictions that planets in this temperature regime are unlikely to possess strong day-to-night temperature contrast^126–128^), M/H = 1.375 (that is, about 20 times solar), C/O ratio of 0.2 and K/O ratio of 0.1. With these atmospheric properties, the data are best explained by the droplet sedimentation model (ScCHIMERA cloud model 3) and inhomogeneous cover. However, the grey + power-law model (ScCHIMERA cloud model 2) with inhomogeneous cover provides a comparable fit to the data. We compare sets of models by computing their Bayes factor and converting to a ‘sigma’ detection significance using the prescription in refs. ^39,40^. Using *R* = 300 data, the homogeneous droplet sedimentation model (model 3) is preferred over homogeneous grey cloud (model 1) at ≳8*σ*, which strongly indicates the non-grey nature of cloud opacity. Meanwhile, the inhomogeneous droplet sedimentation model is preferred over the homogeneous droplet sedimentation model cloud at 5*σ*. This is evidence that, for the same model 3, inhomogeneous cloud coverage is preferred. The inhomogeneous droplet sedimentation model is preferred over all the other tested models across all aforementioned resolutions tested.

We explore the contribution of different chemical species to our reference model by performing the Bayesian inference using the inhomogeneous cloud model 3 and artificially disabling the contribution of a selected chemical species, one at a time. By redoing the Bayesian inference, we are able to compare the Bayesian evidence by computing the Bayes factor and converting to a ‘sigma’ detection significance described above. We detected H_2_O at >30*σ*, K at 6.8*σ* and CO at 3.6*σ*, but no notable detections of Na, CH_4_, CO_2_, HCN and H_2_S. The best-fit metallicity across all models is about 10–30 times solar, the best fit K/O ratio 1–2 times solar and C/O ratio 0.2. Taking the average and standard deviation of the best-fit results for all 20 runs (that is, five models on four data resolutions), we find an average M/H = 19 times solar with a standard deviation of 5 times solar and an average K/O ratio 1.5 times solar with a standard deviation of 0.26 times solar.

### Wavelength sensitivity to inferences

We investigate the dependence of the inferred atmospheric properties on the spectral range of the observations by performing the same Bayesian inferences described above on the spectrum blueward of 2 μm (see Extended Data Fig. 7b). This exercise is repeated on all 20 model–data combinations from ScCHIMERA. With the exception of the solar-to-super-solar K/O ratio, inferences about the atmospheric metallicity, C/O ratio and clouds are primarily driven by the shallower transit depth seen in the *λ* range 2.1–2.3 μm. This wavelength region is that in which the traces of orders 1 and 2 overlap on the detector. To assess the robustness of our results, we explore different data treatments that could affect the final spectrum. First, we find that there are no zeroth-order background contaminants that could be diluting the transit depth in this region. Second, we extract the transmission spectra and fit for dilution between the orders (supreme-SPOON data reduction) and without accounting for the overlap (supreme-SPOON, nirHiss and transitspectroscopy). The evidence for minimal dilution stems from reducing the data through both methods with the same pipeline (supreme-SPOON), which uses the same steps for the entire reduction process along the way, with the exception of fitting and not fitting for dilution. Both techniques yield similar results in the *λ* range 2.1–2.3 μm. We note that the contamination from order 2 into order 1 was previously shown to be between 8 and 12 ppm (ref. ^50^) and is therefore negligible.

We find that, without the data redward of 2 μm, the M/H value is more scattered across models and resolutions with an average metallicity of 61 times solar for the 20 runs and a standard deviation of 28 times solar. On the other hand, the inference on the C/O ratio remains consistently 0.2 across all models and resolutions. Similarly, the K/O ratio remains solar-to-super-solar, with an average of 1.89 times solar and a standard deviation of 0.29 times solar.

These results confirm the necessity for the broad wavelength coverage of NIRISS to constrain the atmospheric metallicity of a planet^8,14,40^. Without the transit depth decrease at 2.1 μm, our models do not exhibit a preference for cloud models 2 and 3 over cloud model 1, nor do they prefer the presence of inhomogeneities in the cloud cover. Without these constraints on the cloud properties, a wide range of metallicities can provide an equally good fit to the observations blueward of 2 μm when combined with different cloud properties, preventing reliable constraints on the metallicity.

The exploration of these models is summarized in Extended Data Fig. 7. The top panel shows the different cloud treatments and their goodness of fit to the data. Overall, models with inhomogeneous cloud cover best explain the data, with the flux-balanced cloud of model 3 giving the lowest *χ*^2^. The bottom panel contrasts the reference model against the results from all cloud models when using data blueward of 2 μm only. Without the information contained in the dip in transit depth at 2.1 μm, all cloud treatments provide an equally good fit and overestimate the transit depth between 2.0 and 2.3 μm.

### K/O ratio inferences

We explore the possibility of constraining the K/O ratio using NIRISS/SOSS. As explained above, across different models and data resolutions, our results indicate that the observations of WASP-39b are best explained by a solar-to-super-solar K/O ratio. To further explore this, we repeat our Bayesian inference for all 20 model–data configurations (five models each at four resolutions) using the observations blueward of 0.8 μm. From high-resolution to low-resolution observations and for all cloud model configurations, we find that all 20 runs prefer models with solar or super-solar K/O ratios for WASP-39b ranging from 1 to 10 times solar. The average across the 20 runs is 2.12 times solar and a standard deviation of 2.33 times solar, with the relatively larger standard deviation resulting from two inferences of highly super-solar K/O ratios of 7 times solar or greater for observations at pixel-level resolution.

Using the reference model atmospheric properties (such as M/H = 1.37, C/O ratio 0.2, full redistribution *f* = 1), we search for the best-fit K/O ratio while simultaneously adjusting the 1-bar radius and the parameters for the inhomogeneous cloud model 3, when only using the observations blueward of 0.8 μm. The best-fit K/O ratio of 0.4 is consistent with the inferences using all the data and the data blueward of 2.0 μm only. This model is shown in Extended Data Fig. 8 in green. For the best-fit cloud parameters and 1-bar radius, we compute a series of K/O ratios spanning sub-solar and super-solar values. We compute the fit of each model to the data using *χ*^2^ statistics. We then convert the resulting *χ*^2^ value to a *P*-value. These *P*-values allow us to estimate the agreement between each model and the data. Our results find that sub-solar K/O ratios are disfavoured to 2*σ*, whereas super-solar values ≳0.7 are disfavoured to 5*σ*.

## Online content

Any methods, additional references, Nature Portfolio reporting summaries, source data, extended data, supplementary information, acknowledgements, peer review information; details of author contributions and competing interests; and statements of data and code availability are available at 10.1038/s41586-022-05674-1.

## Figures and Tables

**Extended Data Table 1:**
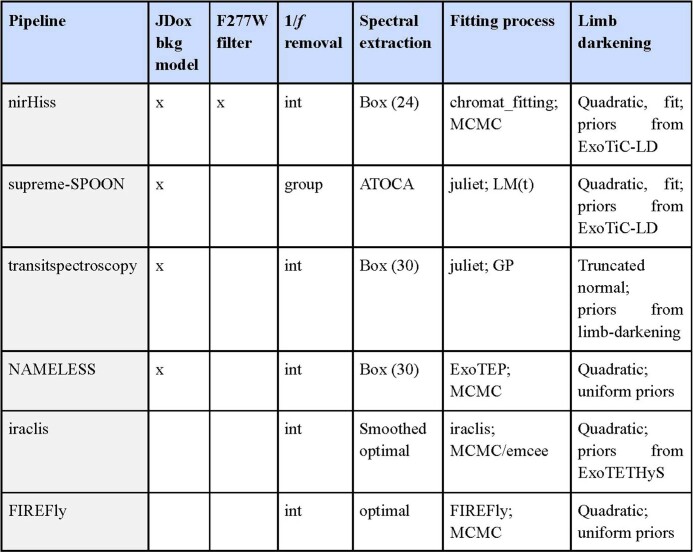
An outline of reduction and fitting pipelines used to produce transmission spectra for WASP-39b with NIRISS/SOSS

The size of the box aperture is listed in parentheses when appropriate. All spectra will be made publicly available.

**Extended Data Table 2:**
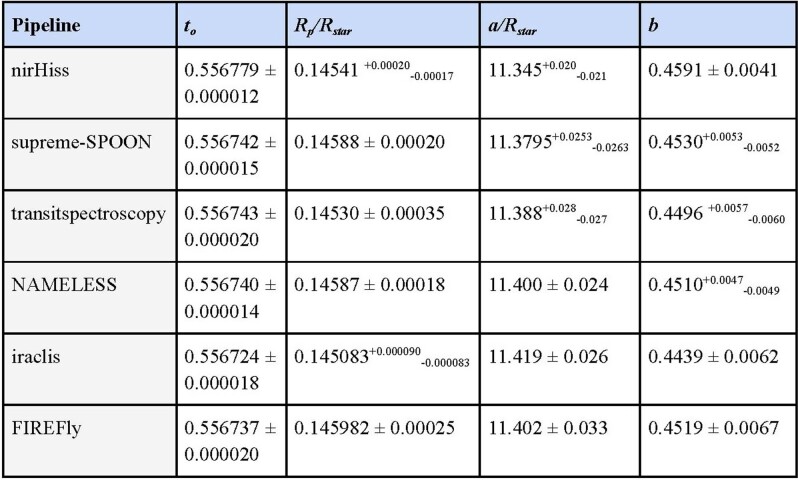
White-light curve best-fit orbital parameters from order 1

Transit time (*t*_0_) is presented with respect to *t*_0_ − 2459787 (BJD). The errors presented on each fit are the 16th and 84th percentile fits to the transit parameter.

## Data Availability

The raw data from this study are publicly available at the Space Telescope Science Institute’s Mikulski Archive for Space Telescopes (https://archive.stsci.edu/). The data used to create all of the figures in this manuscript are freely available on Zenodo and GitHub (Zenodo link: https://github.com/afeinstein20/wasp39b_niriss_paper). All further data are available on request.

## Code availability

The following are open-source pipelines written in Python available either through the Python Package Index (PyPI) or GitHub that were used throughout this work: Eureka! (https://github.com/kevin218/Eureka); nirHiss (https://github.com/afeinstein20/nirhiss); supreme-SPOON (https://github.com/radicamc/supreme-spoon); transitspectroscopy (https://github.com/nespinoza/transitspectroscopy/tree/dev); iraclis (https://github.com/ucl-exoplanets/Iraclis); juliet (https://github.com/nespinoza/juliet); chromatic (https://github.com/zkbt/chromatic); chromatic_fitting (https://github.com/catrionamurray/chromatic_fitting); ExoTiC-LD^13,57^ (https://github.com/Exo-TiC/ExoTiC-LD); ExoTETHyS^125^ (https://github.com/ucl-exoplanets/ExoTETHyS); PICASO^92,93^ (https://github.com/natashabatalha/picaso); Virga^98,99^ (https://github.com/natashabatalha/virga); CHIMERA (https://github.com/mrline/CHIMERA); PyMultiNest (https://github.com/JohannesBuchner/PyMultiNest); MultiNest (https://github.com/JohannesBuchner/MultiNest).

## References

[1] Fischer PD (2016). HST hot-Jupiter transmission spectral survey: clear skies for cool Saturn WASP-39b. Astrophys. J..

[2] Nikolov N (2016). VLT FORS2 comparative transmission spectroscopy: detection of Na in the atmosphere of WASP-39b from the ground. Astrophys. J..

[3] Wakeford HR (2018). The complete transmission spectrum of WASP-39b with a precise water constraint. Astron. J..

[4] Kirk J (2019). LRG-BEASTS: transmission spectroscopy and retrieval analysis of the highly inflated Saturn-mass planet WASP-39b. Astron. J..

[5] Tsiaras A (2018). A population study of gaseous exoplanets. Astron. J..

[6] Fisher C, Heng K (2018). Retrieval analysis of 38 WFC3 transmission spectra and resolution of the normalization degeneracy. Mon. Not. R. Astron. Soc..

[7] Pinhas A, Madhusudhan N, Gandhi S, MacDonald R (2019). H_2_O abundances and cloud properties in ten hot giant exoplanets. Mon. Not. R. Astron. Soc..

[8] Welbanks L, Madhusudhan N (2019). On degeneracies in retrievals of exoplanetary transmission spectra. Astron. J..

[9] Min M, Ormel CW, Chubb K, Helling C, Kawashima Y (2020). The ARCiS framework for exoplanet atmospheres. Modelling philosophy and retrieval. Astron. Astrophys..

[10] Doyon R (2012). The JWST Fine Guidance Sensor (FGS) and Near-Infrared Imager and Slitless Spectrograph (NIRISS). Proc. SPIE.

[11] Stevenson KB (2016). Transiting exoplanet studies and community targets for JWST’s Early Release Science Program. Publ. Astron. Soc. Pac..

[12] Bean JL (2018). The Transiting Exoplanet Community Early Release Science Program for JWST. Publ. Astron. Soc. Pac..

[13] Lecavelier des Etangs A, Pont F, Vidal-Madjar A, Sing D (2008). Rayleigh scattering in the transit spectrum of HD 189733b. Astron. Astrophys..

[14] Line MR, Parmentier V (2016). The influence of nonuniform cloud cover on transit transmission spectra. Astrophys. J..

[15] Ackerman AS, Marley MS (2001). Precipitating condensation clouds in substellar atmospheres. Astrophys. J..

[16] Faedi F (2011). WASP-39b: a highly inflated Saturn-mass planet orbiting a late G-type star. Astron. Astrophys..

[17] Mancini L (2018). The GAPS programme with HARPS-N at TNG. XVI. Measurement of the Rossiter-McLaughlin effect of transiting planetary systems HAT-P-3, HAT-P-12, HAT-P-22, WASP-39, and WASP-60. Astron. Astrophys..

[18] Biazzo K (2022). The GAPS Programme with HARPS-N at TNG. XXXV. Fundamental properties of transiting exoplanet host stars. Astron. Astrophys..

[19] Polanski AS, Crossfield IJM, Howard AW, Isaacson H, Rice M (2022). Chemical abundances for 25 JWST exoplanet host stars with KeckSpec. Res. Not. Am. Astron. Soc..

[20] Lodders K, Palme H, Gail H-P (2009). Abundances of the elements in the Solar System. Landolt Börnstein.

[21] Ahrer, E.-M. et al. Early Release Science of the exoplanet WASP-39b with JWST NIRCam. *Nature*10.1038/s41586-022-05590-4 (2023). 10.1038/s41586-022-05590-4 PMC9946836 36623551

[22] Alderson, L. et al. Early Release Science of the exoplanet WASP-39b with JWST NIRSpec G395H. *Nature*10.1038/s41586-022-05591-3 (2023). 10.1038/s41586-022-05591-3 PMC9946835 36623549

[23] Rustamkulov, Z. et al. Early Release Science of the exoplanet WASP-39b with JWST NIRSpec PRISM. *Nature*10.1038/s41586-022-05677-y (2023). 10.1038/s41586-022-05677-y PMC9946832 36623548

[24] Schneider AD, Bitsch B (2021). How drifting and evaporating pebbles shape giant planets. II. Volatiles and refractories in atmospheres. Astron. Astrophys..

[25] Hands TO, Helled R (2022). Super stellar abundances of alkali metals suggest significant migration for hot Jupiters. Mon. Not. R. Astron. Soc..

[26] Lothringer JD (2021). A new window into planet formation and migration: refractory-to-volatile elemental ratios in ultra-hot Jupiters. Astrophys. J..

[27] Welbanks L (2019). Mass-metallicity trends in transiting exoplanets from atmospheric abundances of H_2_O, Na, and K. Astrophys. J. Lett..

[28] Robertson P, Bender C, Mahadevan S, Roy A, Ramsey LW (2016). Proxima Centauri as a benchmark for stellar activity indicators in the near-infrared. Astrophys. J..

[29] Changeat Q (2022). Five key exoplanet questions answered via the analysis of 25 hot-Jupiter atmospheres in eclipse. Astrophys. J. Suppl. Ser..

[30] Lavvas P, Koskinen T, Yelle RV (2014). Electron densities and alkali atoms in exoplanet atmospheres. Astrophys. J..

[31] Parmentier V, Fortney JJ, Showman AP, Morley C, Marley MS (2016). Transitions in the cloud composition of hot Jupiters. Astrophys. J..

[32] Powell D (2019). Transit signatures of inhomogeneous clouds on hot Jupiters: insights from microphysical cloud modeling. Astrophys. J..

[33] Roman MT (2021). Clouds in three-dimensional models of hot Jupiters over a wide range of temperatures. I. Thermal structures and broadband phase-curve predictions. Astrophys. J..

[34] Demory B-O (2013). Inference of inhomogeneous clouds in an exoplanet atmosphere. Astrophys. J. Lett..

[35] Sing DK (2016). A continuum from clear to cloudy hot-Jupiter exoplanets without primordial water depletion. Nature.

[36] MacDonald RJ, Madhusudhan N (2017). HD 209458b in new light: evidence of nitrogen chemistry, patchy clouds and sub-solar water. Mon. Not. R. Astron. Soc..

[37] Benneke B (2019). A sub-Neptune exoplanet with a low-metallicity methane-depleted atmosphere and Mie-scattering clouds. Nat. Astron..

[38] Barstow JK (2020). Unveiling cloudy exoplanets: the influence of cloud model choices on retrieval solutions. Astrophys. J..

[39] Welbanks L, Madhusudhan N (2021). Aurora: a generalized retrieval framework for exoplanetary transmission spectra. Astrophys. J..

[40] Benneke B, Seager S (2012). Atmospheric retrieval for super-Earths: uniquely constraining the atmospheric composition with transmission spectroscopy. Astrophys. J..

[41] Kreidberg L (2014). A precise water abundance measurement for the hot Jupiter WASP-43b. Astrophys. J. Lett..

[42] Fortney JJ (2013). A framework for characterizing the atmospheres of low-mass low-density transiting planets. Astrophys. J..

[43] Madhusudhan N, Amin MA, Kennedy GM (2014). Toward chemical constraints on hot Jupiter migration. Astrophys. J. Lett..

[44] Mordasini C, van Boekel R, Mollière P, Henning TH, Benneke B (2016). The imprint of exoplanet formation history on observable present-day spectra of hot Jupiters. Astrophys. J..

[45] Shibata S, Helled R, Ikoma M (2020). The origin of the high metallicity of close-in giant exoplanets. Combined effects of resonant and aerodynamic shepherding. Astron. Astrophys..

[46] Lodders K (2003). Solar system abundances and condensation temperatures of the elements. Astrophys. J..

[47] Woitke P (2018). Equilibrium chemistry down to 100 K. Impact of silicates and phyllosilicates on the carbon to oxygen ratio. Astron. Astrophys..

[48] Öberg KI, Murray-Clay R, Bergin EA (2011). The effects of snowlines on C/O in planetary atmospheres. Astrophys. J. Lett..

[49] Eistrup C, Walsh C, van Dishoeck EF (2016). Setting the volatile composition of (exo)planet-building material. Does chemical evolution in disk midplanes matter?. Astron. Astrophys..

[50] Darveau-Bernier A (2022). ATOCA: an algorithm to treat order contamination. Application to the NIRISS SOSS mode. Publ. Astron. Soc. Pac..

[51] Bell, T., Ahrer, E.-M., Brande, J., et al. Eureka! an end-to-end pipeline for JWST time-series observations. *J. Open Source Softw.* **7**, 4503 (2022).

[52] van Dokkum PG (2001). Cosmic-ray rejection by Laplacian edge detection. Publ. Astron. Soc. Pac..

[53] Craig, M. et al. astropy/ccdproc: v1.3.0.post1. 10.5281/zenodo.1069648 (2017).

[54] Radica M (2022). APPLESOSS: A Producer of ProfiLEs for SOSS. Application to the NIRISS SOSS mode. Publ. Astron. Soc. Pac..

[55] Espinoza, N. TransitSpectroscopy. 10.5281/zenodo.6960924 (2022).

[56] Tsiaras A (2016). A new approach to analyzing HST spatial scans: the transmission spectrum of HD 209458 b. Astrophys. J..

[57] Rustamkulov Z, Sing DK, Liu R, Wang A (2022). Analysis of a JWST NIRSpec lab time series: characterizing systematics, recovering exoplanet transit spectroscopy, and constraining a noise floor. Astrophys. J. Lett..

[58] Salvatier J, Wiecki TV, Fonnesbeck C (2016). Probabilistic programming in Python using PyMC3. PeerJ Comput. Sci..

[59] Claret A (2000). A new non-linear limb-darkening law for LTE stellar atmosphere models. Calculations for −5.0 ≤ log[M/H] ≤ +1, 2000 K ≤ T_eff_ ≤ 50000 K at several surface gravities. Astron. Astrophys..

[60] Sing DK (2010). Stellar limb-darkening coefficients for CoRot and Kepler. Astron. Astrophys..

[61] Laginja I, Wakeford H (2020). ExoTiC-ISM: a Python package for marginalised exoplanet transit parameters across a grid of systematic instrument models. J. Open Source Softw..

[62] Gelman A, Rubin DB (1992). Inference from iterative simulation using multiple sequences. Stat. Sci..

[63] Vehtari, A., Gelman, A., Simpson, D., et al. Rank-normalization, folding, and localization: an improved R for assessing convergence of MCMC (with discussion). *Bayesian Anal.* **16**, 667–718 (2021).

[64] Espinoza N, Kossakowski D, Brahm R (2019). Juliet: a versatile modelling tool for transiting and non-transiting exoplanetary systems. Mon. Not. R. Astron. Soc..

[65] Kipping DM (2013). Efficient, uninformative sampling of limb darkening coefficients for two-parameter laws. Mon. Not. R. Astron. Soc..

[66] Wakeford, H. & Grant, D. Exo-TiC/ExoTiC-LD: ExoTiC-LD v2.1 Zenodo release. 10.5281/zenodo.6809899 (2022).

[67] Maciejewski G (2016). New transit observations for HAT-P-30 b, HAT-P-37 b, TrES-5 b, WASP-28 b, WASP-36 b, and WASP-39 b. Acta Astron..

[68] Foreman-Mackey D, Agol E, Ambikasaran S, Angus R (2017). Fast and scalable Gaussian process modeling with applications to astronomical time series. Astron. J..

[69] Espinoza N, Jordán A (2016). Limb darkening and exoplanets – II. Choosing the best law for optimal retrieval of transit parameters. Mon. Not. R. Astron. Soc..

[70] Espinoza N, Jordán A (2015). Limb darkening and exoplanets: testing stellar model atmospheres and identifying biases in transit parameters. Mon. Not. R. Astron. Soc..

[71] Howarth ID (2011). On stellar limb darkening and exoplanetary transits. Mon. Not. R. Astron. Soc..

[72] Benneke B (2019). Water vapor and clouds on the habitable-zone sub-Neptune exoplanet K2-18b. Astrophys. J. Lett..

[73] Mandel K, Agol E (2002). Analytic light curves for planetary transit searches. Astrophys. J..

[74] Kreidberg L (2015). batman: BAsic Transit Model cAlculatioN in Python. Publ. Astron. Soc. Pac..

[75] Foreman-Mackey D, Hogg DW, Lang D, Goodman J (2013). emcee: the MCMC hammer. Publ. Astron. Soc. Pac..

[76] Tsiaras, A. et al. pylightcurve: exoplanet lightcurve model. https://www.ascl.net/1612.018 (2016).

[77] Morello G (2020). ExoTETHyS: tools for exoplanetary transits around host stars. J. Open Source Softw..

[78] Morello G (2020). The ExoTETHyS package: tools for exoplanetary transits around host stars. Astron. J..

[79] Hellier C (2014). Transiting hot Jupiters from WASP-South, Euler and TRAPPIST: WASP-95b to WASP-101b. Mon. Not. R. Astron. Soc..

[80] Virtanen, P. et al. scipy/scipy: SciPy 1.5.3. 10.5281/zenodo.4100507 (2020).

[81] Tremblin P (2015). Fingering convection and cloudless models for cool brown dwarf atmospheres. Astrophys. J. Lett..

[82] Drummond B (2016). The effects of consistent chemical kinetics calculations on the pressure-temperature profiles and emission spectra of hot Jupiters. Astron. Astrophys..

[83] Goyal JM (2018). A library of ATMO forward model transmission spectra for hot Jupiter exoplanets. Mon. Not. R. Astron. Soc..

[84] Goyal JM (2020). A library of self-consistent simulated exoplanet atmospheres. Mon. Not. R. Astron. Soc..

[85] Barber RJ, Tennyson J, Harris GJ, Tolchenov RN (2006). A high-accuracy computed water line list. Mon. Not. R. Astron. Soc..

[86] Yurchenko SN, Tennyson J (2014). ExoMol line lists – IV. The rotation–vibration spectrum of methane up to 1500 K. Mon. Not. R. Astron. Soc..

[87] Tashkun SA, Perevalov VI (2011). CDSD-4000: high-resolution, high-temperature carbon dioxide spectroscopic databank. J. Quant. Spectrosc. Radiat. Transf..

[88] Rothman LS (2010). HITEMP, the high-temperature molecular spectroscopic database. J. Quant. Spectrosc. Radiat. Transf..

[89] Ryabchikova T (2015). A major upgrade of the VALD database. Phys. Scr..

[90] Hauschildt PH, Allard F, Baron E (1999). The NextGen model atmosphere grid for 3000 ≤ *T*_eff_ ≤ 10,000 K. Astrophys. J..

[91] Barman TS, Hauschildt PH, Allard F (2001). Irradiated planets. Astrophys. J..

[92] Lothringer JD, Barman TS (2020). The PHOENIX exoplanet retrieval algorithm and using H^−^ opacity as a probe in ultrahot Jupiters. Astron. J..

[93] Rothman LS (2009). The HITRAN 2008 molecular spectroscopic database. J. Quant. Spectrosc. Radiat. Transf..

[94] Kurucz, R. & Bell, B. Atomic line data, CD-ROM no. 23. Smithsonian Astrophysical Observatory (1995).

[95] McKay CP, Pollack JB, Courtin R (1989). The thermal structure of Titan’s atmosphere. Icarus.

[96] Marley MS, McKay CP (1999). Thermal structure of Uranus’ atmosphere. Icarus.

[97] Batalha NE, Marley MS, Lewis NK, Fortney JJ (2019). Exoplanet reflected-light spectroscopy with PICASO. Astrophys. J..

[98] Mukherjee, S., Batalha, N. E., Fortney, J. J., et al. PICASO 3.0: a one-dimensional climate model for giant planets and brown dwarfs. *Astrophys*. *J.* **942**, 71 (2023).

[99] Polyansky OL (2018). ExoMol molecular line lists XXX: a complete high-accuracy line list for water. Mon. Not. R. Astron. Soc..

[100] Yurchenko SN, Amundsen DS, Tennyson J, Waldmann IP (2017). A hybrid line list for CH_4_ and hot methane continuum. Astron. Astrophys..

[101] Huang X, Gamache RR, Freedman RS, Schwenke DW, Lee TJ (2014). Reliable infrared line lists for 13 CO_2_ isotopologues up to *E*′=18,000 cm^−1^ and 1500 K, with line shape parameters. J. Quant. Spectrosc. Radiat. Transf..

[102] Li G (2015). Rovibrational line lists for nine isotopologues of the CO molecule in the *X*^1^Σ^+^ ground electronic state. Astrophys. J. Suppl. Ser..

[103] Rooney CM, Batalha NE, Gao P, Marley MS (2022). A new sedimentation model for greater cloud diversity in giant exoplanets and brown dwarfs. Astrophys. J..

[104] Mai C, Line MR (2019). Exploring exoplanet cloud assumptions in JWST transmission spectra. Astrophys. J..

[105] Bohren, C. F. & Huffman, D. R. *Absorption and Scattering of Light by Small Particles* (Wiley, 1983).

[106] Arcangeli J (2018). H^−^ opacity and water dissociation in the dayside atmosphere of the very hot gas giant WASP-18b. Astrophys. J. Lett..

[107] Piskorz D (2018). Ground- and space-based detection of the thermal emission spectrum of the transiting hot Jupiter KELT-2Ab. Astron. J..

[108] Mansfield M (2021). A unique hot Jupiter spectral sequence with evidence for compositional diversity. Nat. Astron..

[109] JWST Transiting Exoplanet Community Early Release Science Team (2022). Identification of carbon dioxide in an exoplanet atmosphere. Nature.

[110] Fortney JJ (2005). The effect of condensates on the characterization of transiting planet atmospheres with transmission spectroscopy. Mon. Not. R. Astron. Soc..

[111] Line MR (2013). A systematic retrieval analysis of secondary eclipse spectra. I. A comparison of atmospheric retrieval techniques. Astrophys. J..

[112] Iyer AR, Line MR (2020). The influence of stellar contamination on the interpretation of near-infrared transmission spectra of sub-Neptune worlds around M-dwarfs. Astrophys. J..

[113] Feroz F, Hobson MP, Bridges M (2009). MULTINEST: an efficient and robust Bayesian inference tool for cosmology and particle physics. Mon. Not. R. Astron. Soc..

[114] Buchner J (2014). X-ray spectral modelling of the AGN obscuring region in the CDFS: Bayesian model selection and catalogue. Astron. Astrophys..

[115] Line, M. R. et al. Information content of exoplanetary transit spectra: an initial look. *Astrophys. J.* **749**, 93 (2012).

[116] Richard C (2012). New section of the HITRAN database: collision-induced absorption (CIA). J. Quant. Spectrosc. Radiat. Transf..

[117] Freedman RS (2014). Gaseous mean opacities for giant planet and ultracool dwarf atmospheres over a range of metallicities and temperatures. Astrophys. J. Suppl. Ser..

[118] Azzam Ala’a AA, Tennyson J, Yurchenko SN, Naumenko OV (2016). ExoMol molecular line lists – XVI. The rotation–vibration spectrum of hot H_2_S. Mon. Not. R. Astron. Soc..

[119] Barber RJ (2014). ExoMol line lists - III. An improved hot rotation–vibration line list for HCN and HNC. Mon. Not. R. Astron. Soc..

[120] Kramida, A., Ralchenko, Y., Reader, J. & NIST ASD Team. *NIST Atomic Spectra Database V 5.6* (National Institute of Standards and Technology, 2018).

[121] Allard NF, Spiegelman F, Leininger T, Molliere P (2019). New study of the line profiles of sodium perturbed by H_2_. Astron. Astrophys..

[122] Allard NF, Spiegelman F, Kielkopf JF (2016). K–H_2_ line shapes for the spectra of cool brown dwarfs. Astron. Astrophys..

[123] Gharib-Nezhad E (2021). EXOPLINES: molecular absorption cross-section database for brown dwarf and giant exoplanet atmospheres. Astrophys. J. Suppl. Ser..

[124] Grimm SL (2021). HELIOS-K 2.0 opacity calculator and open-source opacity database for exoplanetary atmospheres. Astrophys. J. Suppl. Ser..

[125] Ohno K, Kawashima Y (2020). Super-Rayleigh slopes in transmission spectra of exoplanets generated by photochemical haze. Astrophys. J. Lett..

[126] Perez-Becker D, Showman AP (2013). Atmospheric heat redistribution on hot Jupiters. Astrophys. J..

[127] Komacek TD, Showman AP (2016). Atmospheric circulation of hot Jupiters: dayside–nightside Temperature differences. Astrophys. J..

[128] Zhang X (2020). Atmospheric regimes and trends on exoplanets and brown dwarfs. Res. Astron. Astrophys..

[129] Bradley, L. et al. astropy/photutils: 1.0.0. 10.5281/zenodo.4044744 (2020).

